# Solubilizing Benzodifuranone-Based Conjugated Copolymers with Single-Oxygen-Containing Branched Side Chains

**DOI:** 10.1021/acsapm.3c02137

**Published:** 2023-12-07

**Authors:** Diego R. Hinojosa, Nathan J. Pataki, Pietro Rossi, Andreas Erhardt, Shubhradip Guchait, Francesca Pallini, Christopher McNeill, Christian Müller, Mario Caironi, Michael Sommer

**Affiliations:** † Institut für Chemie, Technische Universität Chemnitz, Straße der Nationen 62, 09111 Chemnitz, Germany; ‡ Forschungszentrum MAIN, TU Chemnitz, Rosenbergstraße 6, 09126 Chemnitz, Germany; § Center for Nano Science and Technology, Via Rubattino 81, 20134 Milano, Italy; ∥ Department of Physics, Politecnico di Milano, Piazza Leonardo da Vinci 32, 20133Milano ,Italy; ⊥ Department of Materials Science and Engineering, Monash University, Clayton, Victoria 3800, Australia; # Institute Charles Sadron, Université de Strasbourg, Strasbourg F-67000, France; ∇ Department of Chemistry and Chemical Engineering Chalmers University of Technology Göteborg 412 96, Sweden; ○ Department of Materials Science, Università di Milano-Bicocca, via Cozzi 55, 20125 Milan, Italy

**Keywords:** solubility, benzodifuranone polymers, n-type conjugated polymers, branched side chains, branching point variation, organic thermoelectrics

## Abstract

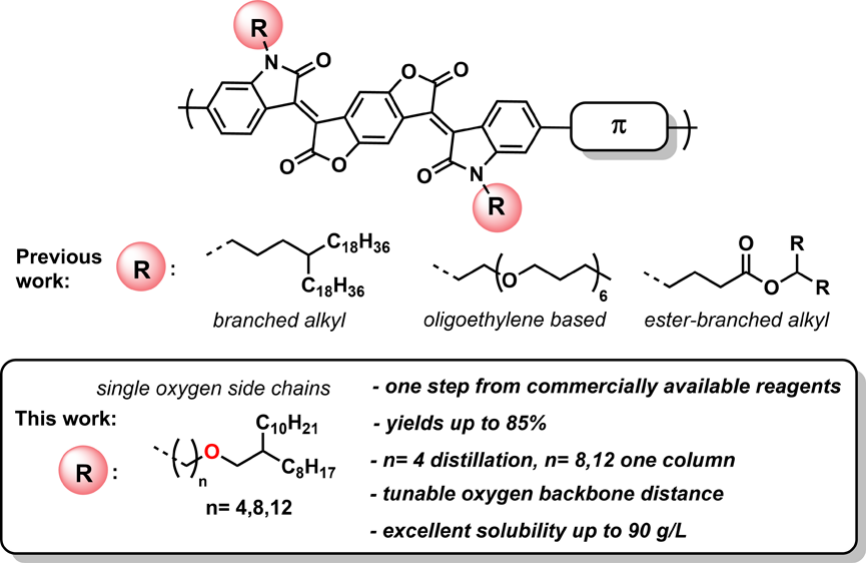

Single-oxygen-containing branched side chains are designed and used to solubilize n-type copolymers consisting of BDF (benzodifuranone), isatin, and thiophene-based units. We present a simple synthetic approach to side chains with varying linker distances between the backbone and the branching point. The synthetic pathway is straightforward and modular and starts with commercially available reagents. The side chains give rise to excellent solubilities of BDF-thiophene copolymers of up to 90 mg/mL, while still being moderate in size (26–34 atoms large). The excellent solubility furthermore allows high molar mass materials. BDF-thiophene copolymers are characterized in terms of optoelectronic and thermoelectric properties. The electrical conductivity of chemically doped polymers is found to scale with molar mass, reaching ∼1 S/cm for the highest molar mass and longest backbone-branching point distance.

## 1 Introduction

Since their discovery, conjugated polymers (CPs) have attracted much attention due to their unique properties.^1,2^ In most cases, CPs comprise two main parts: a π-conjugated backbone, responsible for the optoelectronic properties of the final material and side chains. Side chains are flexible, differently, large pendant groups grafted onto the π-backbone of the polymer.^3^ The tremendous synthetic freedom in the modulation of these groups allows the design of functional side chains that bestow special properties on the macromolecule. For example, introducing ionic groups furnishes conjugated polyelectrolytes that can be processed from water and other polar solvents for application as polymer light-emitting diodes (PLEDs)^4^ and polymer-based photovoltaic cells.^5^ End-chain functionalized side chains, consisting of reactive groups, have also been reported as dormant reactive groups to be further reacted via post-polymerization to achieve different properties.^6−10^ Modification of the electronic properties of the π-conjugated backbone is also possible via side-chain engineering. Directly connecting electron-donating groups^11−13^ or electron-withdrawing groups^14−17^ onto the main chain allows to control the electronic density in the backbone, thus aiding in the modulation of the highest occupied molecular orbital (HOMO) and lowest unoccupied molecular orbital (LUMO) energy levels in the polymer.

Among the most advantageous properties of conjugated polymers is the possibility to process them from solution. Therefore, the use of side chains to induce solubilization is a key and largely exploited aspect, but side chains are also crucial for the ordering of conjugated polymers in the solid state.^18,19^ The most common side chains used for this purpose are alkyl and oligoethylene glycol (OEG) chains, with the latter allowing better solubility in more polar organic solvents. Both types can be found as linear or branched species. Solubility and the propensity to form ordered domains are only some of the properties affected by the choice of the different side chain types, as shown for instance by diketopyrrolopyrrole copolymers.^20,21^ Branched alkyl side chains increase solubility in general.^22^ Their use is commonly required in the case of large backbone structures with a high degree of coplanarity.^23,24^ As the backbone twisting decreases, the π–π interactions between chains increase, which reduces solubility and poses limitations to the parameter space for processing from solution. Introducing a branching point also profoundly affects the final polymer structure and performance, with a longer distance between the conjugated backbone and the branching point reducing sterical hindrance and allowing for better packing, and finally improved performance.^25−28^

Nonsymmetrical branched side chains are customarily prepared from commercially available Gruebert alcohol precursors.^29^ Normally, these alcohols need to be transformed to either an electrophilic reagent that is substituted later by a nucleophile in the monomer as done for instance with diketopyrrolopyrroles (DPP),^30^ or to a nucleophilic amine that reacts with an anhydride for imide-based polymers, as, e.g., naphthalene diimides.^31^ Symmetrically branched side chains on the other hand are tedious to prepare;^32^ their preparation involves multistep synthetic processes, multiple columns, and purification techniques that reduce the overall polymer yield and increase the total cost.^33^ Furthermore, modulation of the branching point in these systems is cumbersome due to their lengthy synthetic pathway.

Benzodifuranone (BDF) is an electron-deficient building block that has most recently been used to prepare self-doping homopolymers with conductivities of up to 6000 S cm^–1^.^34,35^ However, extension and modification of the BDF core, e.g., by conjugation to isatin units, and finally copolymerization with comonomers, is required to modulate *n-*type properties for multiple application scenarios.^36−38^ Conjugated polymers with BDF cores often show intermolecular hydrogen bonding which enables highly planarized structures and close backbone contacts that result in better charge carrier transport properties.^39,40^ BDF copolymers in which the neighbored isatin units are unsubstituted require less synthetic steps but also exhibit modest electrical conductivities of up to 0.26 S cm^–1^ upon doping.^36^ Derivatization of the isatin unit improves electrical conductivities to values of 14 S cm^–1^, but additional synthetic steps are required that add up to the overall cost of the material. Generally, improved properties of BDF-containing polymers commonly correlate with coplanar backbones, which pose a challenge in terms of solubility. The vast majority of studies carried out with BDF polymers have made use of rather large, branched alkyl side chains consisting of a linear C_*x*_ spacer and symmetric, long branches (C_18_).^41^ While providing solubility, the synthesis of such branched alkyl side chains suffers from lengthy synthetic pathways, cumbersome purification, and low overall yields. Moreover, air- and moisture-sensitive Grignard reagents are involved in their preparation, further complicating synthesis and scale-up.^33^ In this context it is noteworthy that a variation of the length of the linear spacer is highly beneficial for the optimization of electron mobility for this class of copolymers, and hence synthetic approaches aiming at simplification of synthetic routes are highly desirable.^42^

For applications involving molecular doping, linear polar side chains of the OEG have been used. However, strong aggregation produces poor miscibility in solution between the polymer and dopant, and phase separation in films, which results in lower electrical conductivity.^43^ Branched alkyl side chains with ester groups have also been presented.^44^ These offer modularity in terms of the side chain length and ester positioning but are susceptible to hydrolysis, limiting long-term stability and preventing their use in greener polymerization methods such as direct arylation polycondensation (DAP) requiring basic conditions.

**Scheme 1:**
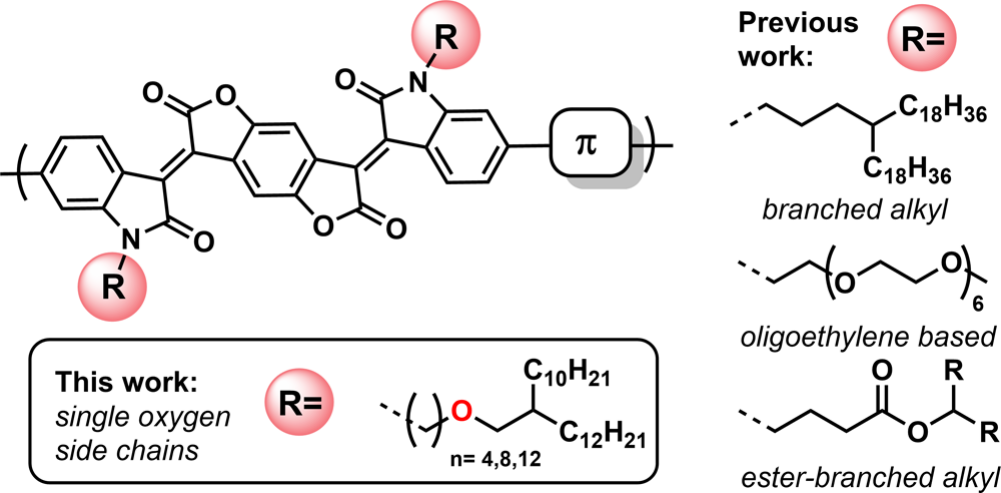
Overview of Side Chain Systems Used for BDF Copolymers and Scope of This Work

The present work introduces single oxygen-containing branched side chains and uses them for solubilizing unsubstituted BDF-containing copolymers (Scheme 1). The side chains can be prepared in one step from commercially available and economic reagents through a simple Williamson etherification reaction and do not require lengthy purification protocols. The availability of different α, ω-dibromo alkyl spacers, and various Guerbert alcohols render this approach ideal for tuning the backbone-oxygen distance as well as the backbone-branching point distance by choosing different starting materials. Thanks to a chemically robust ether moiety, their synthesis is straightforward and highly modular in terms of spacer and branch length, and the side chains render BDF-based copolymers highly soluble. The presented protocol also enables BDF-isatin monomer synthesis on the gram scale. The ether functionality in the side chain provides high flexibility and leads to excellent solubilities of BDF-isatine-thiophene copolymers up to ∼90 mg/mL in *o-*dichlorobenzene (*o-*DCB). Finally, high molar mass copolymers are feasible with conductivities up to 1 S cm^–1^ upon chemical doping, which outperform similar unsubstituted BDF copolymers reported so far.

## 2 Experimental Section

All details regarding starting materials, methods, synthesis of monomers and polymers, and their characterizations are given in the Supporting Information.

## 3 Results and Discussion

### 3.1 Synthesis and Characterization of BDF Copolymers with Single-Oxygen-Containing Side Chains

**Scheme 2:**
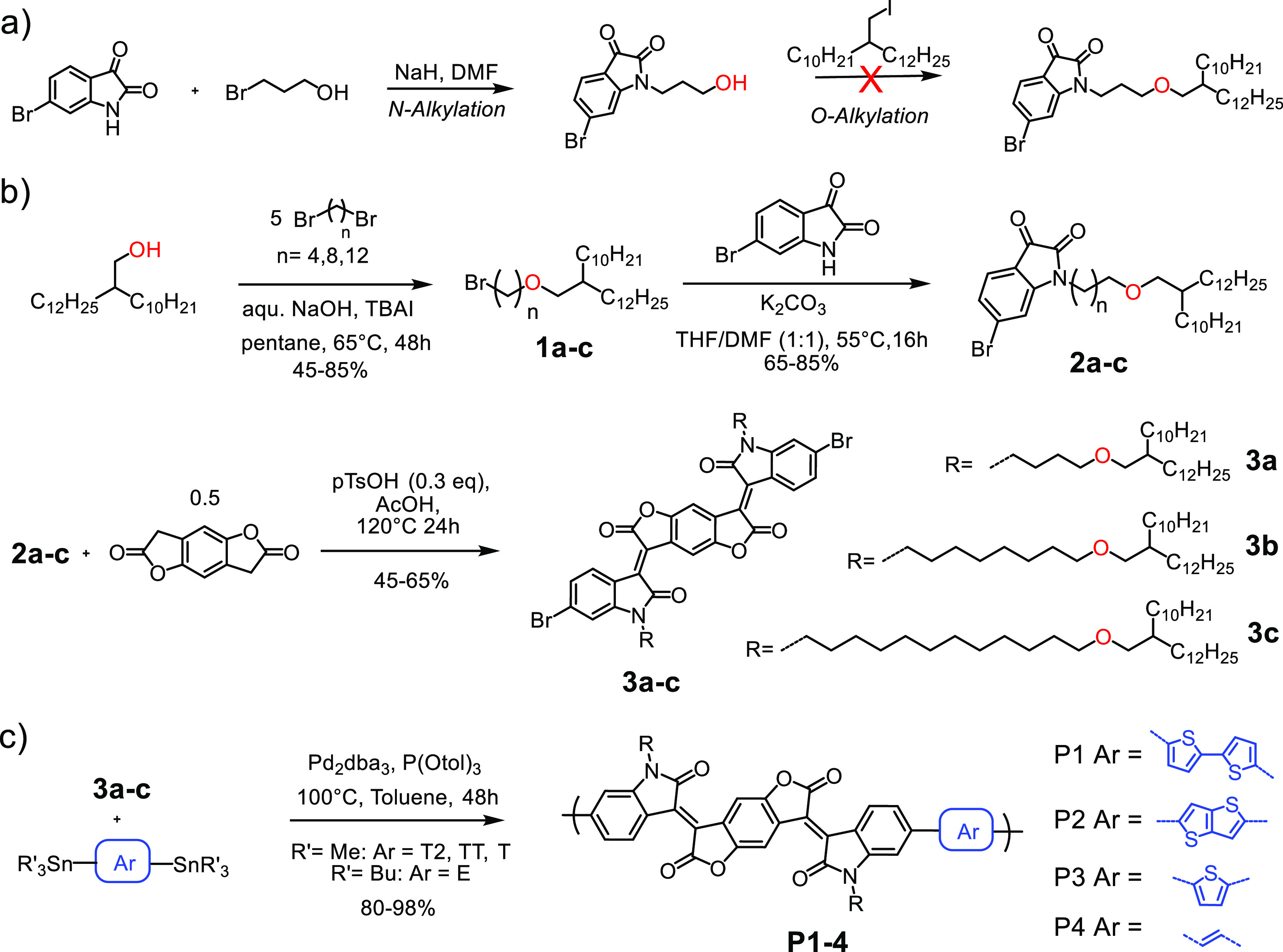
(a) Failed Attempts To Synthesize Single Oxygen Side Chains Attached to Isatin Building Blocks; (b) Successful Route to the Synthesis of Single Oxygen Side Chain Containing Bromoisatins **2a**–**c** and Corresponding Benzodifuranone Monomers **3a**–**c**; (c) Stille Polycondensation of the Different Monomers **3a**–**c** to Get Copolymers **P1–P4**

In order to introduce modular, yet simple, side chains that allow for high solubilities, we first *N*-alkylated bromoisatin with 3-bromo-1-propanol furnishing N-propan-1-ol-6-bromoisatin, following O-alkylation with 1-iodo-2-octyldodecane (Scheme 2a). While the N-alkylation of the isatin proceeded smoothly, the O-alkylation required harsher reaction conditions that led to isatin decomposition. This reduced the yield of the final product and made purification tedious. Furthermore, the necessity of the preparation of the halogenated branched alkyl intermediate also lengthened the overall reaction sequence. A simpler route was then envisaged, in which the side chain was made first, followed by the more straightforward N-alkylation of bromoisatin (Scheme 2b). Starting from an excess of commercially available α–ω-dibromoalkanes and Guerbet^29^ alcohols, the branched halogenated ethers **1a**–**c** could be obtained under Williamson etherification conditions in good yields. Purification was simplified by using a silica plug and distillation in the case of **1a** and a single column in the case of both **1b** and **1c**.

The simplicity of the synthesis protocol shown in Scheme 2b enabled the use of a range of α–ω-dibromoalkanes with varying lengths. Thus, alkylated isatin derivatives **2a**–**c** were obtained in good yields. Finally, BDF-based monomers **3a**–**c** were obtained via acidic aldol condensation of the *N*-alkylated isatins and the benzodifuranone core. All intermediates with side chains were thoroughly characterized by nuclear magnetic resonance (NMR) spectroscopy and mass spectrometry (MS) (see Supporting Information Figures S10–S28). Monomer **3a** was used for comonomer screening under Stille polymerization conditions. This cross-coupling variant is the method of choice since the lactone motif of BDF is prone to hydrolysis under basic conditions.^45^ Variation of the stannylated coupling partner furnished polymer series **P1–P4** (Scheme 2c). The polymers were obtained in good to excellent yields and with high number-average molecular weights *M*_n_ as measured by size exclusion chromatography (SEC) in 1,2,4-trichlorobenzene at 150 °C. Only **P4** with stannylated ethylene as a comonomer exhibited low molar mass resulting from reduced reactivity. The properties of all copolymers are summarized in Table 1.

**Table 1:** Molecular Weights, Dispersities, Yields, and Solubilities of Copolymers **P1**–**P4**

entry	polymer	side chain/comonomer Ar	*M*_n_ [kg/mol]	*Đ*	yield [%]	solubility [mg/mL]
1	**P1a**	4O/T2	n.d.^a^	n.d	97	3
2	**P2a**	4O/TT	n.d.^a^	n.d	98	7
3	**P3a**	4O/T	50.0	2.0	95	89
4	**P3b**	8O/T	55.3	1.8	95	70
5	**P3c**^**55**^	12O/T	54.9	2.5	98	92
6	**P3c**^**47**^	12O/T	46.5	1.9	92	n.d.
7	**P3c**^**46**^	12O/T	46.1	1.7	87	n.d.
8	**P3c**^**42**^	12O/T	42.0	1.5	89	n.d.
9	**P3c**^**28**^	12O/T	28.0	1.5	85	n.d.
10	**P4a**	4O/E	10.5	2.6	80	n.d.

a *M*_n_ could not be determined due to low solubility and difficulties in SEC sample preparation. The superscript in entries 5–9 indicates the *M*_n,SEC_ value of the batch.

### 3.2 Side Chain Alkyl Spacer Length Variation and Electronic Performance

**Figure 1:**
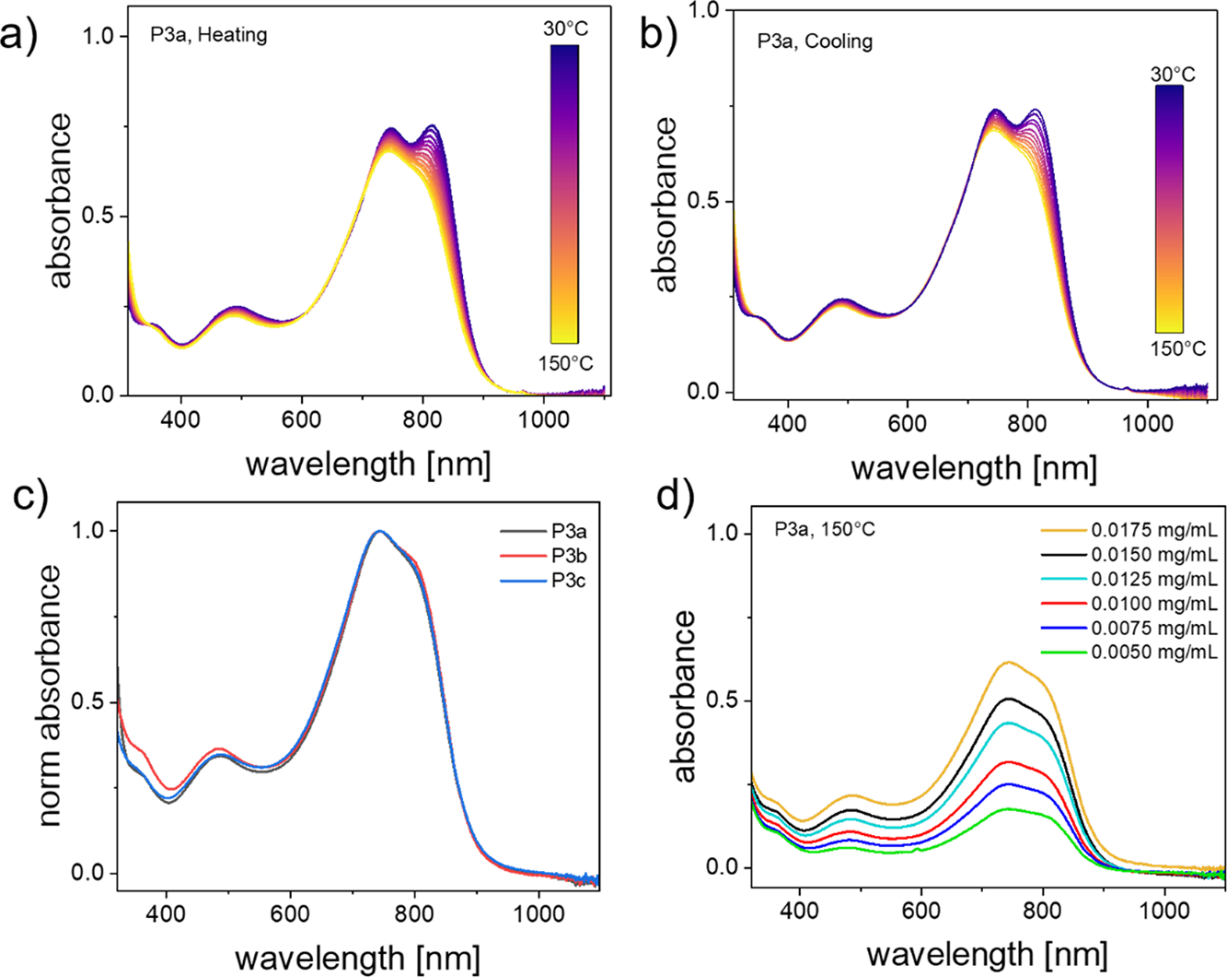
(a) Variable temperature UV–vis spectra of **P3a** (0.02 mg/mL) in *o-*DCB heating from 30 to 150 °C. (b) Variable temperature UV–vis spectra of **P3a** (0.02 mg/mL) in *o-*DCB cooling from 150 to 30 °C. (c) UV–vis spectra of 0.02 mg/mL **P3a**, **P3b** and **P3c** at 150 °C in *o-*DCB. (d) UV–vis spectra of **P3a** at different polymer concentrations in *o-*DCB at 150 °C.

Thiophene was used as a reference comonomer, furnishing a series of copolymers **P3a–c** with side chain and molar mass variation as shown in Table 1. For this series, the ether oxygen is located at distances of 4, 8, or 12 carbons from the backbone. Reasonably high *M*_n_ values around ∼50–55 kg/mol were obtained for all three side chain lengths, indicating that stoichiometry and/or end group degradation may be limiting factors here. The similarity in molecular weights may further result from similar solubilities. We determined the solubility of the polymers quantitatively.^46^ Values of up to 92 mg/mL in *o-*DCB at 150 °C were observed for **P3c**, representing the highest solubility in the polymer series (Figures 1a, S1).

To better understand solubility-dependent aggregation in solution, a variable temperature UV–vis study of **P3a–c** in *o-*DCB was conducted (Figures 1b, S2 and S3). From 30 to 150 °C, the shoulder at ∼820 nm decreased in intensity as more conformations with larger dihedral angles became accessible.^46,47^ Upon cooling the solution from 150 to 30 °C, the original spectrum was restored, indicating a reversible process. A comparison of the optical absorption spectra of the three different polymers **P3a**, **P3b**, and **P3c** revealed only small differences with respect to varying shoulder intensities for **P3b**, in good agreement with the lowest solubility of this copolymer.

The optoelectronic properties of the polymers were characterized by cyclic voltammetry (CV) and steady-state absorbance spectroscopy in *o-*DCB solution and in thin film (Table 2). The predominant electron-withdrawing effect of the BDF monomer dominates the electron affinity of the material. Thus, all copolymers exhibited deep LUMO levels around −4.0 eV measured by CV in solution and in film (Figures S4 and S5). The UV–vis spectra of solutions and thin films revealed a blue shift of the vibronic transitions as the donor strength of the comonomer increased, in good agreement with the push–pull character of the system. Strong aggregation in the *o-*DCB solution can be observed for the thiophene-based polymers **P1–P3** (Figure S6). Aggregation band intensity decreases from **P1** to **P3**. This is expected, as bithiophene and thienothiophene enable larger π stacking areas and more linear backbone geometries compared to thiophene. Although all polymers **P1–4** were found to be soluble in hot *o-*DCB, bithiophene (**P1**) and thienothiophene (**P2**) copolymers were less readily dissolved, yielding gelated mixtures that were difficult to process, even at low concentrations. For this reason, **P3**, with thiophene as the comonomer, was selected as the reference polymer for the study of the electrical and solubility properties.

**Table 2:** Summary of the Optoelectronic Properties of **P1**–**P4**^a^

entry	polymer	λ_max,sol_ [nm]	band gap_sol_ [eV]	λ_max,film_ [nm]	band gap_film_ [eV]	HOMO/LUMO_film_ [eV]
1	**P1a**	860	1.28	843	1.29	–5.47/-4.15
2	**P2a**	831	1.34	825	1.34	–5.51/-4.02
3	**P3a**	812	1.35	815	1.34	–5.55/-4.06
4	**P3b**^**55**^	812	1.36	n.d	n.d	n.d
5	**P3c**^**55**^	812	1.34	n.d	n.d	n.d
6	**P3c**^**46**^	n.d	n.d	n.d	n.d	n.d
7	**P3c**^**42**^	n.d	n.d	n.d	n.d	n.d
8	**P3c**^**28**^	n.d	n.d	n.d	n.d	n.d
9	**P4a**	800	1.29	690	1.43	–4.80/-3.51

a The superscript of the polymers indicates number average molecular weights from SEC *M*_n,SEC_. n.d., not determined.

**Figure 2:**
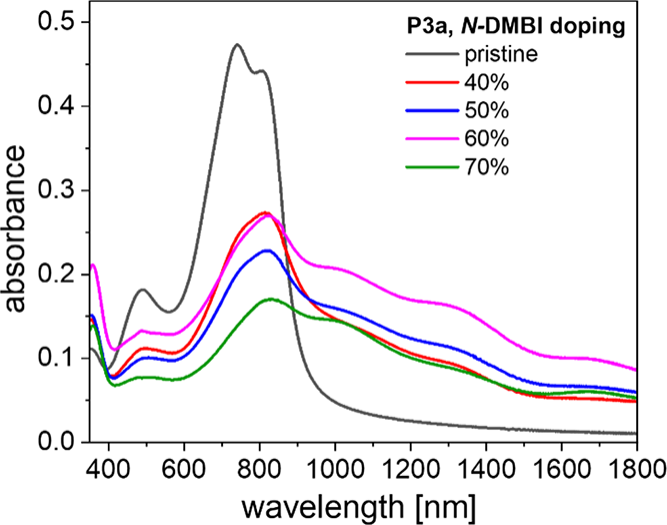
UV–vis-NIR spectra of spin-cast thin films of **P3a** doped with *N*-DMBI.

Well-studied molecular *n*-dopants 4-(1,3-dimethyl-2,3-dihydro-1*H*-benzoimidazol-2-yl)-*N*,*N*-dimethylaniline (*N*-DMBI) and 4-(1,3-dimethyl-2,3-dihydro-1*H*-benzoimidazol-2-yl)-*N*,*N*-diphenylaniline (*N*-DPBI) were used to investigate the *n*-doped characteristics of spin-cast polymer films.^48,49^ A first confirmation of the effective doping with *N*-DMBI and *N*-DPBI is obtained from the spin-casted thin film absorption spectrum of **P3a** coprocessed with *N*-DMBI of varying concentration. Doping levels were determined as molar ratios (MR%) of 40 MR%, 60 MR%, and 80 MR% (Figure 2). The absorbance spectrum of pristine **P3a** is characterized by prominent features at 740 and 810 nm corresponding to the 0–1 and 0–0 vibronic transitions, respectively, where the 0–1 peak shows slightly stronger absorption. Upon addition of *N*-DMBI (Figure 2) the spectra of **P3a** are characterized by a sharp bleaching of both 0–1 and 0–0 features, but there is a stronger bleaching of the 0–1 peak compared to that of the 0–0 peak. At 40 MR% *N-*DMBI doping, the 0–0 absorption is stronger than the 0–1 peak, reversing the relationship from the pristine film. As the doping concentration increases to 80 MR% the 0–1 shoulder almost entirely fades with respect to the 0–0 band. A slight redshift in the absorbance of the 0–0 band can also be seen as doping concentration increases. In addition to the bleaching of the vibronic bands in the visible range, the appearance of broad NIR polaron absorption bands centered around 1020, 1320 and 1690 nm, can be seen. As *N*-DMBI doping concentration increases and bleaching of the vibronic bands intensifies, the intensity of these polaronic bands also increase. Aditionally, the stability under air of doped films of **P3a** was also investigated (Figure S7).

Upon coprocessing **P3a** with *N*-DPBI, the resulting absorption spectra displayed similarities to the *N*-DMBI doped samples, but the intensity of the vibronic bleaching is reduced and the polaronic NIR bands are of weaker appearance (Figure S8). The differences between the doped samples indicate a higher concentration of charged polymer species in the *N*-DMBI-doped samples, which can be linked to an enhanced carrier density. To reinforce this assertion, the electrical conductivity, σ, of the doped thin films was measured and the *N*-DMBI-doped films were found to have a σ_max_ = 0.2 S cm^–1^ while the *N*-DPBI-doped films exhibited a σ_max_ = 0.1 S cm^–1^ at 60 MR%.

**Figure 3:**
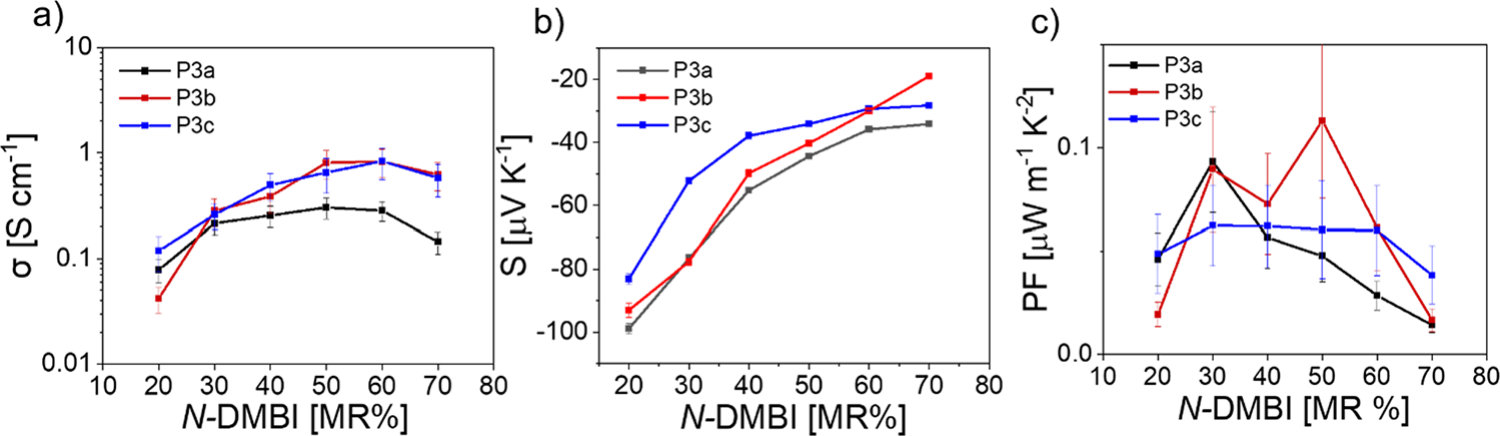
(a) Electrical conductivity of coprocessed thin films of **P3a–c** with respect to *N*-DMBI dopant concentration. (b) Seebeck coefficient of coprocessed thin films **P3a-c** with respect to *N*-DMBI dopant concentration. (c) Power factors of **P3a–c** versus *N*-DMBI dopant concentration calculated from conductivity and Seebeck coefficients. Error bars for electrical conductivity values arise from the experimental error of the film thickness measurement and do not represent a standard deviation.

Due to the superior performance of **P3a** with *N*-DMBI, this dopant was selected to further characterize the electrical conductivity σ, the Seebeck coefficient *S,* and the power factor PF of the full polymer series **P3a–c** (Figures 3, S9). Spin-cast thin films were prepared by coprocessing *N*-DMBI with the three polymers and dopant concentrations ranging from 20 to 70 MR%. Electrical conductivity as a function of increasing dopant concentration followed a similar trend for all of the polymers (Figure 3a). Conductivity increased with increasing dopant concentration of *N*-DMBI from 20 MR% onward, after which a maximum σ_max_ between 50 and 60 MR% appeared. Finally, conductivity decreased again at 70 MR% dopant concentrations. **P3a** exhibited lower values than its longer alkyl spacer counterparts, with a σ_max_ for **P3a** of 0.3 S cm^–1^ at a dopant concentration of 50 MR%. Conductivities for the longer alkyl spacer counterparts **P3b** and **P3c** were twice as high compared to those of **P3a**, reaching σ_max_ values of 0.82 and 0.83 S cm^–1^, respectively, at dopant concentrations of 60 MR%. This represents an almost 3-fold increase in conductivity compared to similar polymers reported in the literature.^36^

The Seebeck coefficients of the polymer-dopant blends were characterized using a custom-built setup that employed a quasi-static measurement method (Figure 3b, details are reported in the Supporting Information).^50^ The Seebeck coefficients of the polymers were found to have the largest magnitude at the lowest dopant concentration, 20 MR%, with *S*_P3a_ = −98.9 μV K^–1^ being the largest of the three polymers. As dopant concentration increases, the magnitude of the Seebeck coefficient decreases for all three polymers, which agrees well with the empirical relationship between thermovoltage and charge carrier concentrations seen throughout the literature.^51,52^ At dopant concentrations greater than 40 MR%, the order of Seebeck coefficients was *S*_P3a_ > *S*_P3b_, *S*_P3c_, while σ_P3a_ < σ_P3b_, σ_P3c_, indicating that the increased length of the alkyl spacer improves electrical conductivity while also slightly eroding the thermovoltage.

The power factor PF = σ*S*^2^ was calculated for each sample and showed a trend similar to the electrical conductivity, namely that polymers **P3b** and **P3c** with longer distances between backbone and oxygen yielded higher PFs compared to **P3a**. That being said, **P3b**, the polymer with the intermediate spacer length, demonstrated the best thermoelectric performance out of the series with a PF_P3b_ > 0.1 μW m^–1^ K^–2^. The exact nature of the thermoelectric improvement with respect to spacer length is not known from these results alone, but it is logical to argue that an increasing side chain length allows the polymer matrix to host a larger number amount of dopant cations.^53^ Additionally, oxygen in the side chain provides two electron lone pairs that may interact with dopant cations.

### 3.3 Investigation of Polymer Morphologies in Pristine and Doped States

**Figure 4:**
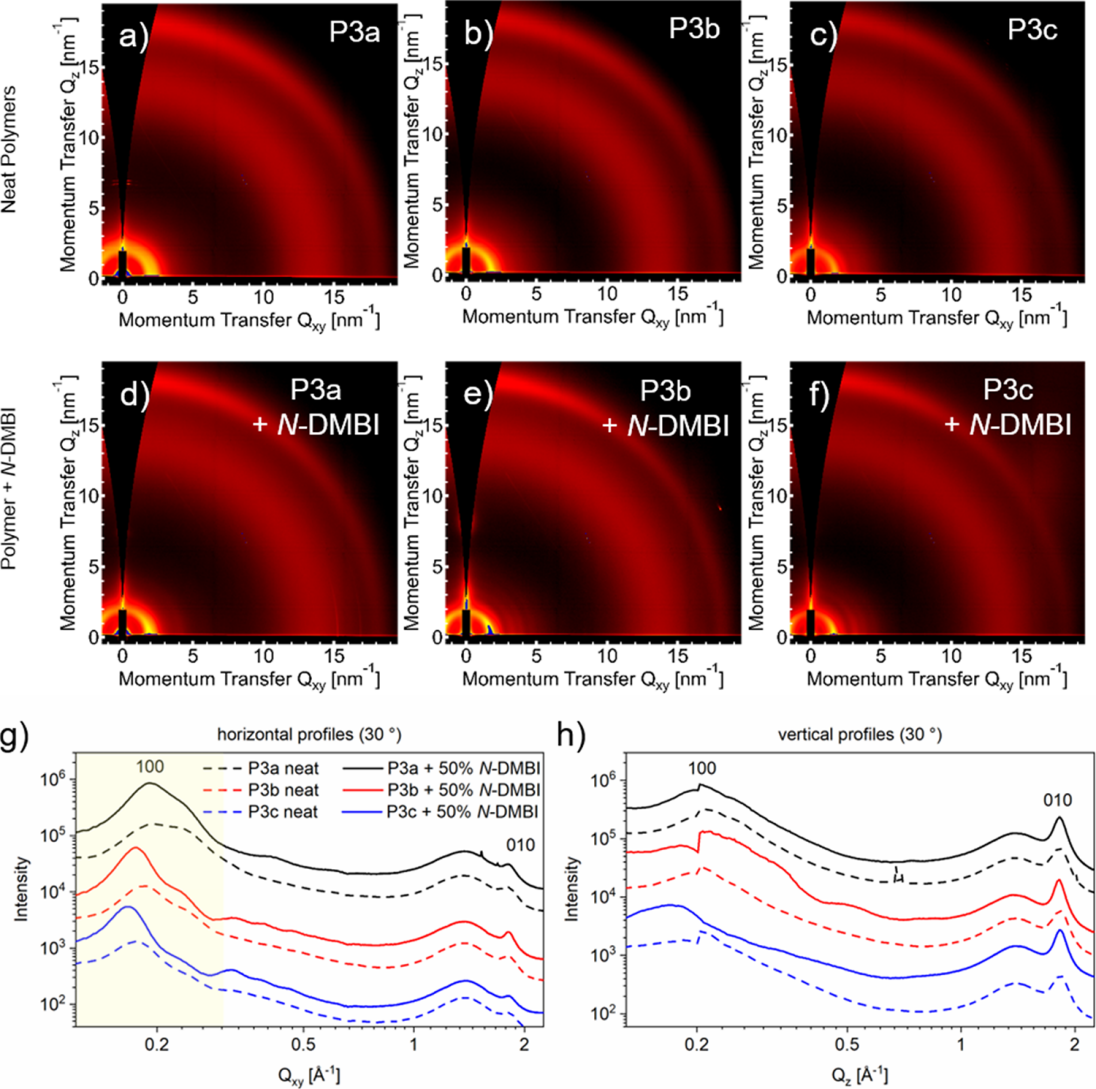
2D GIWAXS images of the neat polymers **P3a**, **P3b** and **P3c** (a–c) and the respective blends with 50% *N-*DMBI (d–f) and the respective line profiles. Profiles were extracted in horizontal (g) and vertical (h) directions and are depicted in dashed lines for the neat films and solid lines for the *N-*DMBI blends. The 100 side-chain stacking peaks that change significantly during doping and side chain substitution are highlighted in the horizontal profiles. The line cuts were offset for clarity.

To correlate changes in electrical performance and morphology as well as evaluate the microstructure of blends of the polymers with the dopant *N-*DMBI, grazing incidence wide-angle scattering (GIWAXS) experiments were conducted (Figure 4). From the observed 2D scattering patterns (Figure 4a–f), the polymers can be considered to be weakly ordered as a result of slightly curved backbones. For all polymers, side-chain stacking peaks can be observed predominantly in-plane at ∼0.2 Å^–1^, while a well-defined 010 π-stacking peak can be seen predominantly out-of-plane at ∼1.75 Å^–1^. The observations indicate a preferential “face-on” packing of chains within a morphology characterized by a high degree of mosaicity, that is, a broad distribution of the orientation of crystallites. A prominent amorphous halo is also observed 1.4 Å^–1^ suggesting a sizable amorphous fraction. The position of the 100 peaks systematically shifts to a lower *Q* value going from **P3a** to **P3b** to **P3c**, which corresponds to an increasing side-chain stacking distance, consistent with the increase in the length of the alkyl side chain spacer (Figure 4g, dashed lines). This correlation matches the expected behavior, as an increase in side-chain volume consequently should lead to an increased distance of the side-chain domains. The position of the 010 peaks, in contrast, does not change between the three materials (Figure 4h, dashed lines), indicating that the π–π stacking behavior is not affected by the modification of the side chains.

Structural changes upon doping are visible both in the 2D scattering patterns and in the line profiles. When the pristine polymers are compared to the respective blend (Figure 4g, h, solid lines), a shift to smaller *Q*-values or a larger real space distance is observable in the 100 peaks, while the 010 signals remain unaffected. We ascribe this to a preferential accumulation of *N-*DMBI in the side-chain regions. Such an effect has been observed for polymers without OEG-substituted polymers before.

The lamellar expansion, however, is relatively small. Considering the high amount of *N-*DMBI added, we conclude that the majority of dopant is located in the amorphous domains of the polymer, which are not represented as sharp signals in the scattering patterns. If the relative shifts of the 100 peaks upon doping of the three polymers are compared, a relatively small change is observable for **P3a**, while **P3b** and **P3c** both experience a larger lamellar volume expansion. This would suggest that a higher amount of *N-*DMBI is incorporated into the crystalline domains of **P3b** and **P3c**, which is reflected in their superior conductivity (cf. Figure 4a). Interestingly, the scattering features appear to become sharper with doping, suggesting that the inclusion of the dopant leads to an increase in structural order, which is unusual. We also note there are additional peaks at low *Q* close to the 100 peak that are difficult to index, suggesting either the presence of a polymorph or a more complicated packing geometry.

### 3.4 Correlation of Molar Mass and Conductivity

**Figure 5:**
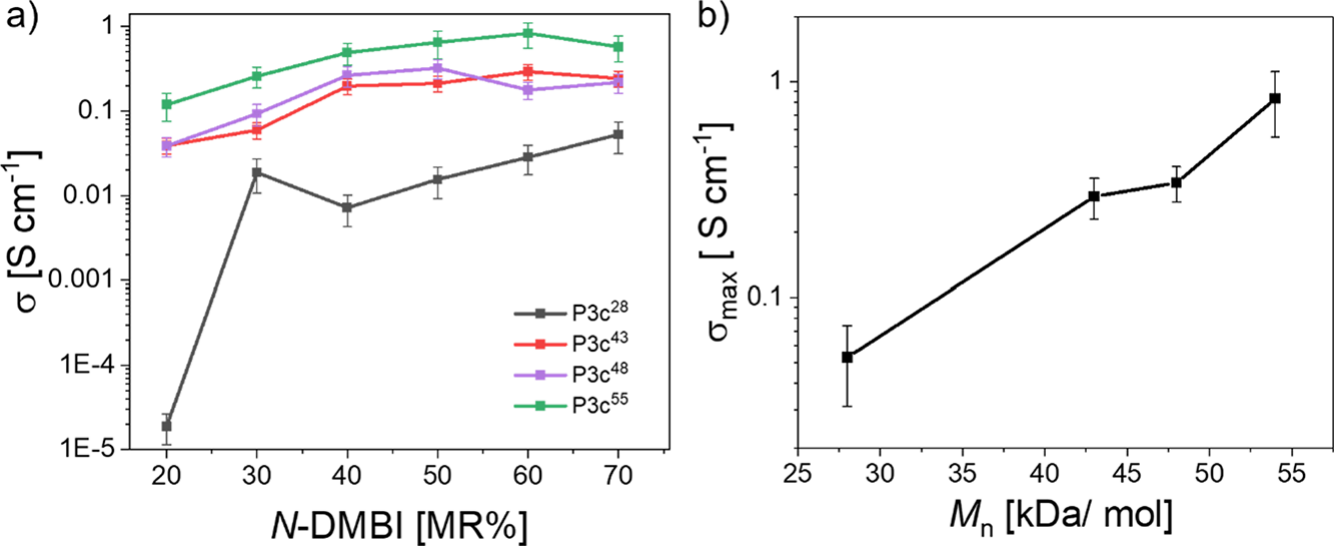
(a) Electrical conductivity of **P3c** thin films versus *N*-DMBI dopant concentration for four different *M*_n_. (b) Electrical conductivity versus *M*_n_ at a doping concentration of *N*-DMBI = 60 MR%. Error bars for electrical conductivity values arise from the experimental error of the film thickness measurement and do not represent a standard deviation.

As the ether side chains induce a high solubility and therefore enable the synthesis of longer chains, we further evaluate the relationship between molar mass and conductivity. Since polycondensation is governed by the Carothers equation, different *M*_n_ values are achieved by a stochiometric imbalance of the BDF-based comonomer (Figures 5, S2). For each molecular weight obtained, conductivities were determined depending on varying doping levels of 20% < MR < 70%, showing similar trends. The sample with the lowest molar mass stood out and showed very low conductivity for low levels of doping, which increased by 3 orders of magnitude to 10^–2^ S/cm.

Clearly, with **P3c**^**28**^ reaching a σ_max_ of 0.05 S cm^–1^ only at high *N-*DMBI concentrations of 70 MR%, a certain threshold molar mass must be reached (Figure 5a). For all larger molecular weights probed, conductivities spanned around 1 order of magnitude. Upon comparison of conductivities of the four polymers at 60 MR%, **P3c**^**43**^ and **P3c**^**46**^ exhibited both σ_max_ ≈ 0.35 S cm^–1^, while for **P3c**^**55**^ a σ_max_ = 0.83 S cm^–1^ is measured, highlighting the importance of a high solubility for the preparation of high molar mass materials (Figure 5b). To the best of our knowledge, the conductivity obtained from doped **P3c**^**55**^ is the highest reported for BDF copolymers featuring nonfunctionalized isatin units, demonstrating that simple building blocks can achieve competitive performance when solubility allows long chains to form.

## 4 Conclusions

In summary, the work presented here introduces aliphatic side chains containing a single ether functionality for solubilizing conjugated polymers with BDF-isatin cores. The side chains are simple to make; their synthesis is scalable, and the resulting copolymers with thiophene as a comonomer exhibit excellent solubilities of up to 92 mg/mL in common organic solvents. The high solubilities allow for the preparation of a series of copolymers with different molar masses to explore molecular weight-property relationships. Here, the largest molar mass sample delivers the highest conductivity, with values of σ ≈ 1 S cm^–1^ after *n*-doping, highlighting the importance of solubility and molar mass control. Such electrical conductivities are superior compared to similar reported copolymers having unsubstituted BDF-isatin cores. GIWAXS analysis of the morphology of the copolymers reveals a high degree of “face-on” packing, as well as a preferential location of the dopant cations in the amorphous regions, along with an increase in the structural order upon doping. The presented side chains can easily be used for a variety of other copolymers where branching point variation is of interest and where solubility limits molar mass.

## Supporting Information Available

All synthetic procedures, characterization data (NMR spectroscopy and mass spectrometry), and descriptions of experimental methods; additional figures, schemes, and tables; NMR spectra, SEC curves, UV–vis spectra, cyclic voltammograms, conductivity data, Seebeck coefficients, and power factors (DOCX)
