# Supplementary material for: Solubilizing Benzodifuranone-Based Conjugated Copolymers with Single-Oxygen-Containing Branched Side Chains
Source: ACS Appl Polym Mater. 2023 Dec 7;6(1):457–65. doi: 10.1021/acsapm.3c02137 (PMC10788869; doi:10.1021/acsapm.3c02137)
Supplement: Supplementary file 1 — ap3c02137_si_001.docx [file ap3c02137_si_001.docx]

Supporting Information

Solubilizing Benzodifuranone-based Conjugated Copolymers with Single Oxygen Containing Branched Side Chains

Diego R. Hinojosa^a,b^, Nathan J. Pataki^c,d^, Pietro Rossi^c,d^, Andreas Erhardt^e^, Shubhradip Guchait^f^, Francesca Pallini^h^, Christopher McNeill^e^, Christian Müller^g^, Mario Caironi^c*^, and Michael Sommer^a,b*^

^a^ Technische Universität Chemnitz, Institut für Chemie, Straße der Nationen 62, 09111 Chemnitz, Germany

^b^ Forschungszentrum MAIN, TU Chemnitz, Rosenbergstraße 6, 09126 Chemnitz, Germany

^c^ Center for Nano Science and Technology, Via Rubattino 81, 20134 Milano, Italy

^d^Department of Physics, Politecnico di Milano, P.zza Leonardo da Vinci 32, Milano 20133, Italy

^e^Department of Materials Science and Engineering, Monash University, Clayton, Victoria 3800, Australia

^f^ Institute Charles Sadron, Université de Strasbourg, Strasbourg F-67000, France

^g^ Department of Chemistry and Chemical Engineering Chalmers University of Technology Göteborg 412 96, Sweden

^h^ Department of Materials Science, Università di Milano-Bicocca, via Cozzi 55, 20125 Milan, Italy

Corresponding Authors: [*michael.sommer@chemie.tu-chemnitz.de](mailto:*michael.sommer@chemie.tu-chemnitz.de)

*[mario.caironi@iit.it](mailto:mario.caironi@iit.it)

TABLE OF CONTENTS

1. General Materials and Methods………………………………………….S3
2. Experimental Procedures………………………………………………...S6
3. Additional Figures……………………………………………………...S14
4. NMR Spectra…………………………………………….......................S20

5 References………………………………………………………….......S31

**1 General Materials and Methods**

*Chemicals*. All chemicals, reagents and solvents were purchased from commercial sources and used as received unless otherwise noted. Solvents used for column chromatography were distilled under reduced pressure before use. Dimethylformamide (DMF) and tetrahydrofuran (THF) were dried one week over molecular sieves 4 Å.

*NMR Spectroscopy*. NMR spectra were recorded on an Avance NEO 600 FT spectrometer (^1^H: 600 MHz, ^13^C: 150.9 MHz). The spectra were referenced to the residual solvent peaks (chloroform: δ (^1^H) = 7.26 ppm, δ (^13^C) = 77.16 ppm). Analysis was executed with Mestrelab Mestrenova v11.0.4. Peak assignments were supported by COSY, HSQC and HMBC experiments.

*High resolution mass spectra (HRMS)* were obtained from a Bruker Trapped Ion Mobility Spectrometry (tims) time-of-flight (TOF) mass spectrometer in APCI+ mode in 60µM solution in MeCN and MeCN:MeOH mixtures (v : v = 7 : 3).

*Size exclusion chromatography (SEC).* SEC of polymers was performed on an Agilent PL-GPC 220 integrated high-temperature GPC/SEC system in 1,2,4-trichlorobenzene at 150 °C using relative calibration with polystyrene standards.

*Cyclic voltammetry (CV).* CV measurements were performed with a scan rate of 50 mV·s −1 at RT under an argon atmosphere using a PalmSens4 potentiostat, a Ag/Ag+ pseudo-reference electrode, and a platinum wire as counter electrode. Ferrocene was used for calibration, and a value of −4.80 eV for the half-wave potential of the Fc/Fc+ couple was used for determining the HOMO and LUMO energy levels. The error on the HOMO/LUMO energy levels is estimated to be at least ±0.10 eV.^1^ For measurements in solution, a glassy carbon working electrode and NBu4PF6 (0.1 M) as electrolyte in dry o-DCB were used. Polymer solutions were measured at a concentration of about 0.15 g/L and monomer solutions at 0.05 mM. For film measurements, an ITO-coated float glass (from Prazisions ̈ Glas & Optik GmbH (pgo), sheet resistance ≤7 Ω·sq−1 ) as working electrode and NBu4PF6 (0.1 M) as electrolyte in dry acetonitrile were used. Thin films were prepared by spin coating (30 s @ 1000 rpm, 30 s @ 3000 rpm) polymer solutions (2.5 g/L in *o-*DCB) onto the ITO electrodes.

*Doped thin film sample preparation.* Borosilicate Corning glass (low alkali, 1737F) substrates were cut into 1.5 cm × 1.5 cm squares. Metallic contacts were patterned on the substrates through a shadow mask by thermal evaporation (MB-ProVap-3) depositing a 3 nm Cr adhesion layer and a thick 50 nm Au layer on top of the Cr layer. Each electrode was 3 mm × 1.5 cm and the interelectrode distance was 5 mm. A stock solution of each polymer in toluene (Sigma-Aldrich) was prepared with a concentration of 7 g L^−1^, to ensure complete dissolution it was kept stirring at 60 °C overnight. Since all doping was conducted through a co-processing method, the same solvent, toluene, was used to prepare stock solutions of *N*-DMBI and *N*-DPBI (Sigma-Aldrich) were prepared at a concentration of 7 g L^−1^. To perform co-processing doping, aliquots of dopant and polymer stock solutions were mixed at corresponding amounts to reach the selected doping concentration. The dopant concentration is expressed in MR%, which is calculated as the ratio between the number of dopant moles over that of polymer repeat units, to allow a better comparison with data available in the literature. Finally, toluene was added to adjust all the aliquots to the same polymer concentration. The glass substrates (both patterned and non-patterned) were cleaned sequentially in deionized water, acetone and 2-propanol (Sigma-Aldrich) submerged in an ultrasonic bath for 10 min each. Following, the substrates were exposed to a O_2_ plasma (Femto Diener electronic) at 100 W for 10 min. The thin films were cast via spin-coating technique inside a N_2_-filled glovebox (MBraun), the deposition parameters were 600 rpm for 40 s followed by 1000 rpm for 10 s and finally 3000 rpm for 3 s. The samples were then thermally annealed on a hotplate for 1 h and at a temperature of 180 °C. Thickness of the films changed depending on the polymer (P3a-c) and *M_n_.* Films spin-cast from P3a had a thickness in the range of 55 ± 5 nm, films spin-cast from P3b had a thickness in the range of 40 ± 5 nm, and films spin-cast from P3c had a thickness in the range of 35 ± 5 nm. The thickness of all the films was determined using a mechanical profilometer (Alpha-step IQ, KLA Tencor).

*UV-vis Spectroscopy*. All UV-vis spectra were measured in transmission. UV-vis spectra in solution were measured at 25 °C on a Cary 60 UV-vis (Agilent Technologies). High Temperature UV/Vis spectra were measured using 0.02 mg/mL solutions of polymers in *o*-DCB in sealed glass cuvettes. The samples were heated using a sample holder from Agilent and 10°C/min heating rate from 30°C to 150°C, with a stabilization time of 5 min for each temperature.

Thin film absorption spectra were recorded on a Flame-S UV-vis spectrometer from Ocean Optics with an integration time of 3 ms and 666 scans to average, using OceanView 1.5.2 software. All UV–vis–NIR absorption spectra of the pristine and doped thin film samples were acquired in air at 25 °C using a double beam Perkin Elmer l1050 spectrophotometer in the 350–1800 nm range.

*Quantitative solubility determination.* For each polymer, six different solutions with varying concentrations from 0.005 mg/mL up to 0.0175 mg/mL were prepared. The UV/vis spectra of these solutions were recorded at 150°C in *o*-DCB and the maximum absorbance of each curve was collected. This data was used to plot a calibration curve. To determine the maximum solubility of each polymer, a saturated solution of the polymer in *o*-DCB at 150°C was prepared by adding polymer until aggregates that did not dissolve any longer become visible. At this point, this hot solution was quickly filtered through a 0.45 μm PTFE filter, and a known volume of the saturated solution was diluted with *o*-DCB by a known dilution factor so that the final solution concentration was within the range of the calibration curve. Finally, the unknown solution UV/Vis spectrum is measured at 150°C and the maximum absorbance is interpolated in the previously generated curve. This value is then multiplied by the known dilution factor to yield the final total polymer concentration in the saturated solution.

*Grazing-incidence small-angle X-ray scattering (GIWAXS).* GIWAXS on films coated on silicon substrates was performed in vacuum at RT on the SAXS/WAXS beamline of the Australian Synchrotron with a photon energy of 15 keV.^2^The setup included a Pilatus 2M detector, which was used to record 2D scattering patterns. Each image consists of three superimposed acquisitions with different relative sample-detector positions at identical distances to obtain gapless scattering data. A silver behenate reference standard was used to calibrate the sample-to-detector distance. The sample and detector were placed in a vacuum chamber to reduce air scatter. The measurements were taken as a function of the angle of incidence, with data shown at an angle of incidence near the critical angle that maximized scattering intensity from the sample. Data evaluation and wedge correction were conducted using a modified Nika software package in IgorPro 8.38.^3^ The 1-dimenstional *Q*-profiles shown are sector cuts covering an azimuthal angle of χ = 75°–105° for the cuts in the vertical direction and χ = 0°–15° as well as χ = 165°–180° for the cuts in the horizontal direction.

*Electrical conductivity measurements.* The *I*–*V* characteristics were collected in the current saturation regime employing a two-point contact configuration by means of a probe-station (Wentworth Laboratories) connected to a semiconductor device parameter analyzer (Agilent B1500A). Measurements were performed at room temperature (RT) in a N_2_-filled glovebox. Forward and backward scans were performed to exclude the presence of hysteresis. The electrical conductivity values were subsequently calculated, considering the geometrical parameters, from the resistance values extrapolated from the *I*–*V* curves.

*Seebeck coefficient measurements.* The in-plane Seebeck coefficient measurements were performed using a custom setup built for thin films characterization and described in detail in the work of Beretta et al.^4^ The measurements were conducted at RT and under vacuum conditions (10^−4^ mbar) to reduce convection phenomena and to avoid samples oxidation. Due to the instrumentation limitations only thin film samples with an electrical resistance below 10 MΩ could be measured.

*Ambient stability measurements.* The ambient stability of two P3a thin films spin-cast from an *o*-DCB and toluene solution were tested. Both P3a solutions had a concentration of 7 g L^−1^ and the *N*-DMBI solution was prepared with the same solvent and concentration to match the polymer stock solutions. The doped aliquots and thin films were prepared in the same manner as described above. After annealing, baseline *I*–*V* characteristics were collected at RT in a N_2_-filled glovebox. The thin film samples were then removed from the glovebox and *I*–*V* characteristics were collected in ambient atmosphere at RT at regular intervals. The electrical conductivity values were subsequently calculated, showing the drop in electrical conductivity for both samples with respect to air exposure time. The film spin-cast from *o*-DCB had a measured thickness of 20 ± 5 nm while the toluene film had a measured thickness of 55 ± 5 nm. The difference in thickness resulted in different degradation rates, but neither film exhibited long-term stability. Upon completion of the test, films were returned to the N_2_-filled glovebox, annealed at 180 °C for 1-2 hrs and remeasured in the glovebox.

**2 Experimental Procedures**

**Synthesis of 1a**

**
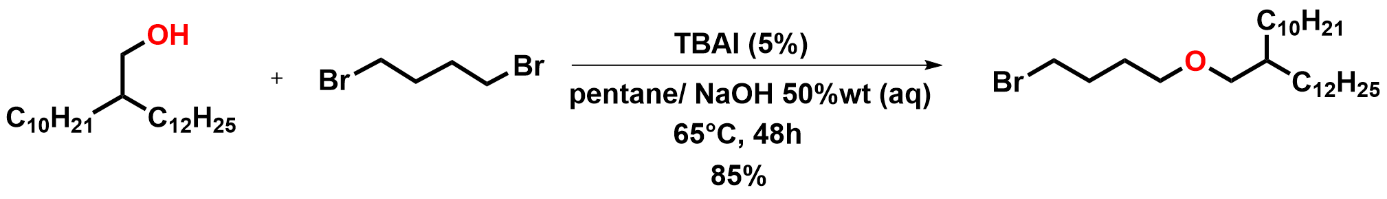
**

In a 250 mL round bottom flask 2-decyl-1-tetradecanol (20.00 g, 56.39 mmol, 1.0 eq), 1.4 dibromobutane (61.5 g, 285.0 mmol, 5.0 eq) and tetrabutyl ammonium iodide (1.04 g, 2.72 mmol, 0.05 eq) were dissolved in 70 mL of pentane. After this, 30 mL of freshly prepared 50 wt.-% NaOH solution were added to the mixture to form a biphasic system. The mixture was refluxed at 65 °C for 48 h under strong stirring. After 48 h, the mixture was cooled down to room temperature, diluted with pentane and extracted with water (3x 50 mL) and brine (3x 50 mL). The organic layer was collected and the solvent removed under vacuum to yield a colorless oil. The crude oil was directly transferred to a silica plug that was washed with copious amounts of pentane. The organic solvent was removed under reduced pressure to yield a colorless oil that was transferred to a 100 mL round bottom flask. This oil was subjected to a high vacuum distillation at 100 °C for 1 h to remove excess of 1,4-dibromobutane. Finally, the residue in the distillation flask was collected as the title compound as a colorless oil (23.6 g, 85% ).

^1^H NMR (600.1 MHz, CDCl_3_) δ / ppm 3.37 (t, *^3^J_HH_* = 6.85 Hz, 2 H, -CH_2_O-DT), 3.34 (t, ^3^*J_HH_* = 6.26 Hz, 2 H, BrCH_2_-), 3.19 (d, *^3^J_HH_* = 6.27 Hz, 2 H, -O-CH_2_-CHR2), 1.88 (p, *^3^J_HH_* = 7.26 Hz 2 H, −CH_2_CH_2_O-DT), 1.64 (p, *^3^J_HH_* = 7.06 Hz, 2 H, BrCH_2_CH_2_-), 1.46 (br, 1 H, -O-CH_2_-CHR2), 1.19 (br, 40 H, DT_internal_), 0.81 (t, *^3^J_HH_* = 7.06 Hz 6 H, DT_terminal_);

^13^C{^1^H} NMR (150.9 MHz, CDCl3) δ / ppm 74.3 (CH_2_O-DT), 69.9 (-O-CH_2_-CHR2), 38.2 (O-CH_2_-CHR2), 33.8-22.5 (DT_internal_), 14.1 (DT_terminal_).

**Synthesis of 1b**

**
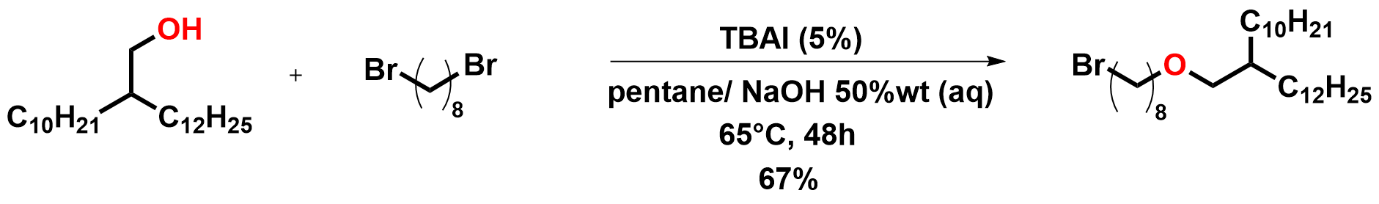
**

In a 250 mL round bottom flask 2-decyl-1-tetradecanol (5 g, 16.74 mmol, 1.0 eq), 1.8 dibromooctane (22.78 g, 83.74 mmol, 5.0 eq) and tetrabutyl ammonium iodide (0.27 g, 0.84 mmol, 0.05 eq) were dissolved in 40 mL of pentane. After this, 15 mL of freshly prepared 50 wt.-% NaOH solution were added to the mixture to form a biphasic system. The mixture was refluxed at 65 °C for 48 h under strong stirring. After 48 h, the mixture was cooled down to room temperature, diluted with pentane and extracted with water (3x 50 mL) and brine (3x 50 mL). The organic layer was collected and the solvent removed under vacuum to yield a colorless oil that was purified by column chromatography using petrol ether:CHCl_3_ ramping from 0 % to 30 % CHCl_3_ (6.12 g, 67% ).

^1^H NMR (600.1 MHz, CDCl_3_) δ / ppm 3.37 (t, *^3^J_HH_* = 6.85 Hz, 2 H, -CH_2_O-OD), 3.34 (t, ^3^*J_HH_* = 6.26 Hz, 2 H, BrCH_2_-), 3.20 (d, *^3^J_HH_* = 6.03 Hz, 2 H, -O-CH_2_-CHR2), 1.91-1.12 (m, 52 H, -(CH_2_)_8_-/DT_internal_), 0.81 (t, *^3^J_HH_* = 7.11 Hz, 6 H, DT_terminal_).

^13^C{^1^H} NMR (150.9 MHz, CDCl3) δ / ppm 74.0 (CH_2_O-OD), 71.0 (-O-CH_2_-CHR2), 38.2 (O-CH_2_-CHR2), 33.8-22.5 (DT_internal_), 14.1 (DT_terminal_).

**Synthesis of 1c**

In a 250 mL round bottom flask 2-decyl-1-tetradecanol (10.65 g, 29.75 mmol, 1.0 eq), 1.12 dibromododecane (26.0 g, 120 mmol, 4.0 eq) and tetrabutyl ammonium iodide (1.50 g, 1.48 mmol, 0.05 eq) were dissolved in 40 mL of pentane. After this, 15 mL of a freshly prepared 50 wt.-% NaOH solution were added to the mixture to form a biphasic system. The mixture was refluxed at 65 °C for 48 h under strong stirring. After 48 h, the mixture was cooled down to room temperature, diluted with pentane and extracted with water (3x 50 mL) and brine (3x 50 mL). The organic layer was collected and the solvent removed under vacuum to yield a colorless oil that was purified by column chromatography using petrol ether:CHCl_3_ ramping from 0% to 30% CHCl_3_ (7.88 g, 44% ).

^1^H NMR (600.1 MHz, CDCl_3_) δ / ppm 3.32 (t, *^3^J_HH_* = 6.85 Hz, 2 H, -CH_2_O-DT), 3.28 (t, ^3^*J_HH_* = 6.26 Hz, 2 H, BrCH_2_-), 3.18 (d, *^3^J_HH_* = 6.03 Hz, 2 H, -O-CH_2_-CHR2), 1.80-1.1 (m, 56 H, -(CH_2_)_8_-/DT_internal_), 0.81 (t, *^3^J_HH_* = 7.11 Hz, 6 H, DT_terminal_).

^13^C{^1^H} NMR (150.9 MHz, CDCl3) δ / ppm 74.2 (CH_2_O-DT), 69.9 (-O-CH_2_-CHR2), 38.2 (O-CH_2_-CHR2), 33.8-22.7 (DT_internal_), 14.1 (DT_terminal_).

**Synthesis of 2a**

**
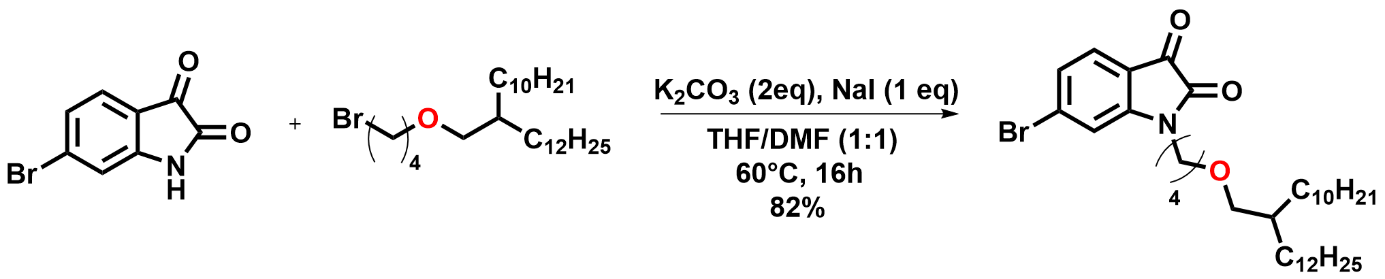
**

In a 250 mL round bottom flask 6-bromoisatin (1.37 g, 6.06 mmol, 1.0eq), K_2_CO_3_, (1.67 g, 12.12 mmol, 2.0 eq), NaI (0.90 g, 6.06 mmol, 1.0 eq) and **1a** (2.63 g, 6.06 mmol, 1.0 eq) were dissolved in 50 mL of a 1:1 mixture of THF/DMF. The mixture was heated for 16 h at 60°C under stirring. The mixture was poured into water (200 mL) and extracted with ethyl acetate (4x 50 mL). The organic fractions were collected, washed with brine (3x 50 mL) and dried with anhydrous Mg_2_SO_4._ The solvent was removed under reduced pressure to yield an orange oil that was purified using column chromatography using petrol ether: ethyl acetate (6:1) (2.40 g, 82 %).

^1^H NMR (600.1 MHz, CDCl_3_) δ / ppm 7.37 (d, *^3^J_HH_* = 7.94 Hz, 1 H, -CH_(4)_), 7.19 (dd *^3^J_HH_* = 7.94 Hz, *^4^J_HH_* = 1.94 Hz, 1 H, CH_(5)_), 7.04 (d, *^4^J_HH_* = 1.94 Hz, 1 H, CH_(1)_), 3.67 (t, *^3^J_HH_* = 7.33 Hz, 2 H, N-CH_2_-), 3.37 (t, *^3^J_HH_* = 6.09 Hz, 2 H, -CH_2_-O-DT), 3.20 (d, *^3^J_HH_* = 5.91 Hz, 2 H, -O-CH_2_-CHR2), 1.72 (p, *^3^J_HH_* = 7.26 Hz, 2 H, −CH_2_CH_2_O-DT),1.57 (p, *^3^J_HH_* = 6.17 Hz, 2 H, NCH_2_CH_2_-), 1.48 (br, 1 H, -O-CH_2_-CHR2), 1.19 (br, 40 H, DT_internal_), 0.81 (t, *^3^J_HH_* = 7.06 Hz 6 H, DT_terminal_)

^13^C{^1^H} NMR (150.9 MHz, CDCl3) δ / ppm 182.4 (C=O_carbonyl_), 158.0 (C=O_amide_), 151.3 (C_1_), 133.3 (C_Br_), 126.8 (C_4_), 126.3 (C_5_), 116.2 (C_3_), 113.9 (C_2_) (CH_2_O-DT), 74.4 (CH_2_O-DT), 70.1 (-O-CH_2_-CHR2), 40.3 (O-CH_2_-CHR2), 33.8-22.5 (DT_internal_), 14.1 (DT_terminal_).

**Synthesis of 2b**


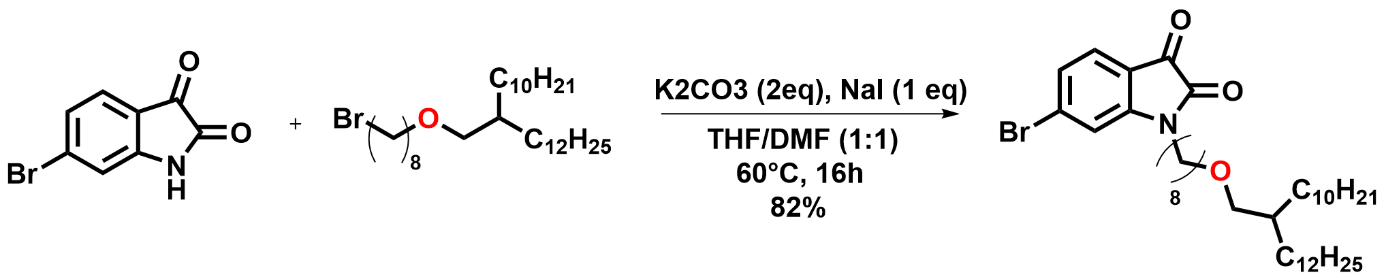


In a 250 mL round bottom flask 6-bromoisatin (1.50 g, 6.63 mmol, 1.0 eq), K_2_CO_3_, (1.83 g, 13.27 mmol, 2.0 eq), NaI (0.99 g, 6.63 mmol, 1.0 eq) and **1b** (3.62g, 6.63 mmol, 1.0eq) were dissolved in 50 mL of a 1:1 mixture of THF/DMF. The mixture was heated for 16 h at 60 °C under stirring. After this time, the crude was poured into water (200 mL) and extracted with ethyl acetate (4x 50 mL). The organic fractions were collected, washed with brine (3x 50 mL) and dried with anhydrous Mg_2_SO_4._ The solvent was removed under reduced pressure to yield an orange oil that was purified using column chromatography with petrol ether: ethyl acetate (6:1) (3.57g, 78%).

^1^H NMR (600.1 MHz, CDCl_3_) δ / ppm 7.38 (d, *^3^J_HH_* = 7.97 Hz, 1 H, -CH_(4)_), 7.19 (d *^3^J_HH_* = 7.94 Hz, 1 H, CH_(5)_), 6.99 (s, 1 H, CH_(1)_), 3.62 (t, *^3^J_HH_* = 7.67 Hz, 2 H, N-CH_2_-), 3.29 (t, *^3^J_HH_* = 6.14 Hz, 2 H, -CH_2_-O-DT), 3.18 (d, *^3^J_HH_* = 5.96 Hz, 2 H, -O-CH_2_-CHR2), 1.80-1.1 (m, 52 H, -(CH_2_)_8_-/DT_internal_), 0.81 (t, *^3^J_HH_* = 7.11 Hz, 6 H, DT_terminal_).

^13^C{^1^H} NMR (150.9 MHz, CDCl3) δ / ppm 182.4 (C=O_carbonyl_), 158.0 (C=O_amide_), 151.3 (C_1_), 133.3 (C_Br_), 126.8 (C_4_), 126.3 (C_5_), 116.2 (C_3_), 113.9 (C_2_) (CH_2_O-DT), 74.4 (CH_2_O-DT), 70.1 (-O-CH_2_-CHR2), 40.3 (O-CH_2_-CHR2), 33.8-22.5 (DT_internal_), 14.1 (DT_terminal_).

**Synthesis of 2c**


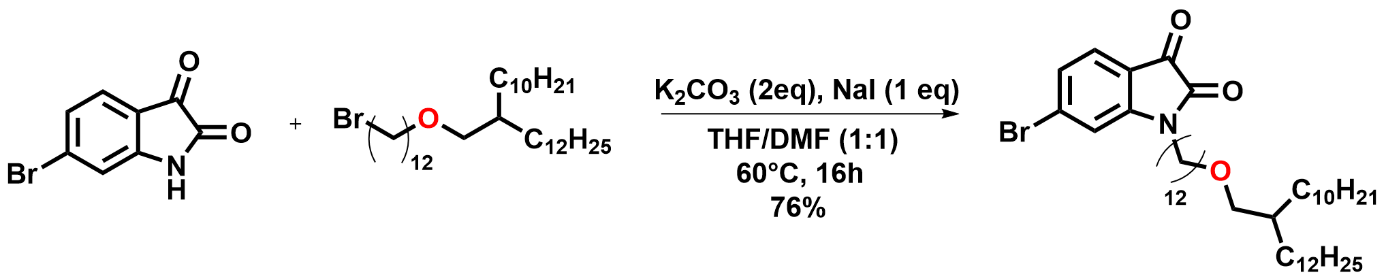


In a 250 mL round bottom flask 6-bromoisatin (1.50 g, 6.63 mmol, 1.0 eq), K_2_CO_3_, (1.83 g, 13.27 mmol, 2.0 eq), NaI (0.99 g, 6.63 mmol, 1.0 eq) and **1c** (4.95 g, 6.63 mmol, 1.0 eq) were dissolved in 50 mL of a 1:1 mixture of THF/DMF. The mixture was heated for 16 h at 60 °C under stirring. After this time, the crude was poured into water (200 mL) and extracted with ethyl acetate (4x 50 mL). The organic fractions were collected, washed with brine (3x 50 mL) and dried with anhydrous Mg_2_SO_4._ The solvent was removed under reduced pressure to yield an orange oil that was purified using column chromatography with petrol ether: ethyl acetate (6:1) (3.76g, 76%).

^1^H NMR (600.1 MHz, CDCl_3_) δ / ppm 7.38 (d, *^3^J_HH_* = 7.97 Hz, 1 H, -CH_(4)_), 7.19 (d *^3^J_HH_* = 7.94 Hz, 1 H, CH_(5)_), 6.99 (s, 1 H, CH_(1)_), 3.62 (t, *^3^J_HH_* = 7.67 Hz, 2 H, N-CH_2_-), 3.29 (t, *^3^J_HH_* = 6.14 Hz, 2 H, -CH_2_-O-DT), 3.18 (d, *^3^J_HH_* = 5.96 Hz, 2 H, -O-CH_2_-CHR2), 1.80-1.1 (m, 56 H, -(CH_2_)_8_-/DT_internal_), 0.81 (t, *^3^J_HH_* = 7.11 Hz, 6 H, DT_terminal_).

^13^C{^1^H} NMR (150.9 MHz, CDCl3) δ / ppm 182.4 (C=O_carbonyl_), 158.0 (C=O_amide_), 151.3 (C_1_), 133.3 (C_Br_), 126.8 (C_4_), 126.3 (C_5_), 116.2 (C_3_), 113.9 (C_2_) (CH_2_O-DT), 74.4 (CH_2_O-DT), 70.1 (-O-CH_2_-CHR2), 40.3 (O-CH_2_-CHR2), 33.8-22.5 (DT_internal_), 14.1 (DT_terminal_).

**Synthesis of 3a**

**
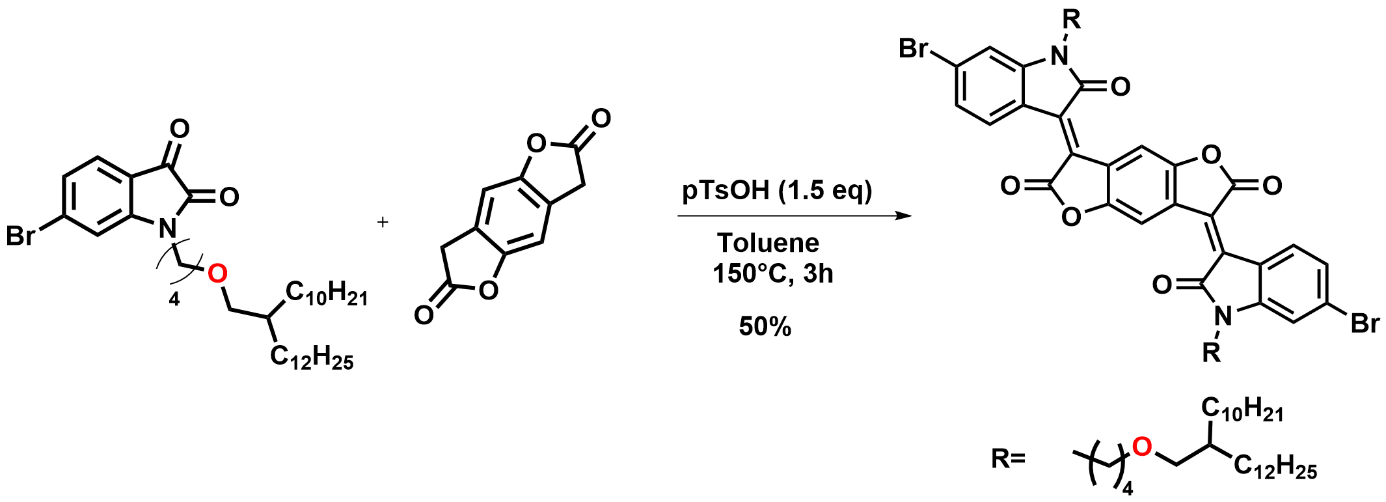
**

In a 250 mL round bottom flask **2a** (2 g, 3.15 mmol, 1.0 eq), benzo[1,2-b:4,5-b’]difuran-2,6(3H,7H)-dione, (0.30 g, 1.57 mmol, 0.5 eq), and p-toluenesulfonic acid (0.88 g, 4.72 mmol, 1.5 eq) were added and the flask subjected to three evacuation/refill cycles with argon. The solids were dissolved in 80 mL of anhydrous toluene and the flask was equipped with a Dean-Stark apparatus. The mixture was refluxed under vigorous stirring for 3 h at 150 °C, during this time the color changed from bright orange to black. After this time the crude mixture was evaporated to dryness, the residue dissolved in chloroform and the organic phases washed three times with water (3x 50 mL) and brine (3x 50 mL). The organic layer was dried and concentrated under reduced pressure, and the residue was purified by column chromatography using petrol ether: dichloromethane (1:1) to yield a sticky black/green solid (1.12 g, 50 %).

^1^H NMR (600.1 MHz, CDCl_3_) δ / ppm 8.95 (s, 1H, CH_BDF_), 8.78 (d, *^3^J_HH_* = 8.73 Hz, 1 H, -CH_(4)_), 7.09 (d *^3^J_HH_* = 8.73 Hz, 1 H, CH_(5)_), 6.91 (s, 1 H, CH_(1)_), 3.70 (t, *^3^J_HH_* = 7.30 Hz, 2 H, N-CH_2_-), 3.37 (t, *^3^J_HH_* = 6.09 Hz, 2 H, -CH_2_-O-DT), 3.20 (d, *^3^J_HH_* = 5.91 Hz, 2 H, -O-CH_2_-CHR2), 1.72 (p, *^3^J_HH_* = 7.26 Hz, 2 H, −CH_2_CH_2_O-DT),1.57 (p, *^3^J_HH_* = 6.17 Hz, 2 H, NCH_2_CH_2_-), 1.48 (br, 1 H, -O-CH_2_-CHR2), 1.19 (br, 40 H, DT_internal_), 0.81 (t, *^3^J_HH_* = 7.06 Hz 6 H, DT_terminal_)

^13^C{^1^H} NMR (150.9 MHz, CDCl3) δ / ppm 167.0 (C=O_amide_), 151.3 (C=O_BDF_), 146.8 (C_2isatin)_, 135.5 (C=C_isatin_), 131,5 (C_4isatin_),129.1 (C_Br_) 126.8 (C=C_BDF_), 126.4 (C_qBDF_), 125.6 (C_5isatin_), 119.7 (C_3isatin_), 111.8(CH_BDF_) 111.0 (C_1isatin_), 74.4 (CH_2_O-DT), 70.1 (-O-CH_2_-CHR2), 38.3 (O-CH_2_-CHR2), 32.1-22.7 (DT_internal_), 14.2 (DT_terminal_).

MALDI-TOF (m/z) (% relative intensity, ion): [M]^+^ calculated: 1423.69, observed: 1423.95.

**Synthesis of 3b**


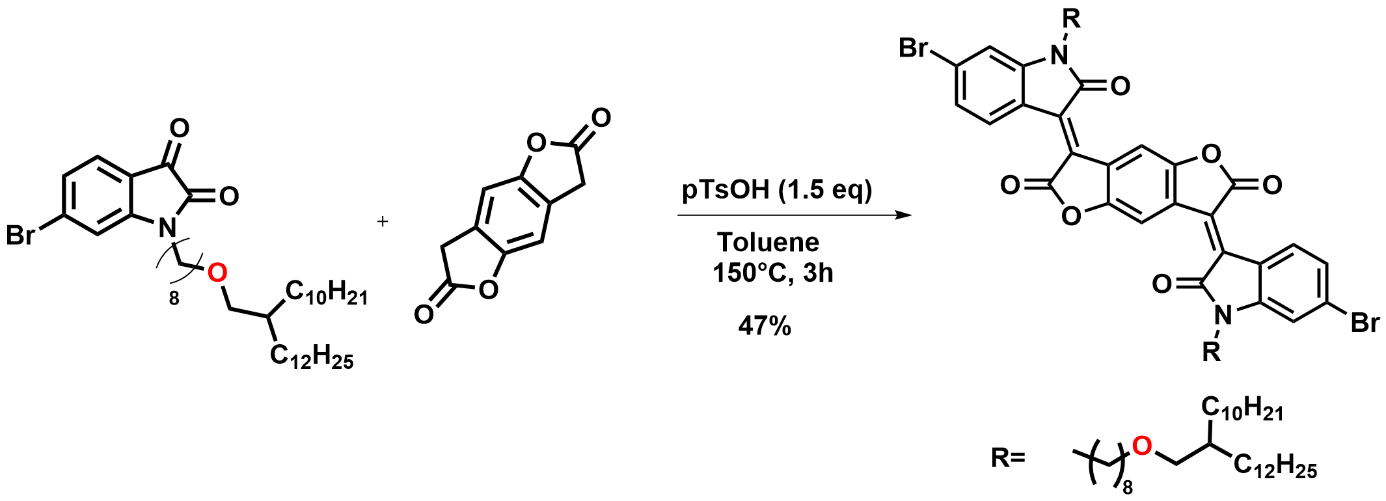


In a 250 mL round bottom flask **2b** (2 g, 2.89 mmol, 1.0 eq), benzo[1,2-b:4,5-b’]difuran-2,6(3H,7H)-dione , (0.27 g, 1.44 mmol, 0.5 eq), and p-toluenesulfonic acid (0.82 g, 4.33 mmol, 1.5 eq) were added and the flask subjected to three evacuation/refill cycles with argon. The solids were dissolved in 80 mL of anhydrous toluene and the flask was equipped with a Dean-Stark apparatus. The mixture was refluxed under vigorous stirring for 3 h at 150 °C, during this time the color changed from bright orange to black. After this time the crude mixture was evaporated to dryness, the residue dissolved in chloroform and the organic phases washed three times with water (3x 50 mL) and brine (3x 50 mL). The organic layer was dried and concentrated under reduced pressure and the residue was purified by column chromatography using petrol ether: dichloromethane (1:1) to yield a sticky black/green solid (1.04g, 47%)

^1^H NMR (600.1 MHz, CDCl_3_) δ / ppm 8.95 (s, 1H, CH_BDF_), 8.78 (d, *^3^J_HH_* = 8.73 Hz, 1 H, -CH_(4)_), 7.09 (d *^3^J_HH_* = 8.73 Hz, 1 H, CH_(5)_), 6.91 (s, 1 H, CH_(1)_), 3.70 (t, *^3^J_HH_* = 7.30 Hz, 2 H, N-CH_2_-), 3.62 (t, *^3^J_HH_* = 7.67 Hz, 2 H, N-CH_2_-), 3.29 (t, *^3^J_HH_* = 6.14 Hz, 2 H, -CH_2_-O-DT), 3.18 (d, *^3^J_HH_* = 5.96 Hz, 2 H, -O-CH_2_-CHR2), 1.80-1.1 (m, 52 H, -(CH_2_)_8_-/DT_internal_), 0.81 (t, *^3^J_HH_* = 7.11 Hz, 6 H, DT_terminal_).

^13^C{^1^H} NMR (150.9 MHz, CDCl3) δ / ppm 167.0 (C=O_amide_), 151.3 (C=O_BDF_), 146.8 (C_2isatin)_, 135.5 (C=C_isatin_), 131,5 (C_4isatin_),129.1 (C_Br_) 126.8 (C=C_BDF_), 126.4 (C_qBDF_), 125.6 (C_5isatin_), 119.7 (C_3isatin_), 111.8(CH_BDF_) 111.0 (C_1isatin_), 74.4 (CH_2_O-DT), 70.1 (-O-CH_2_-CHR2), 38.3 (O-CH_2_-CHR2), 32.1-22.7 (DT_internal_), 14.2 (DT_terminal_).

MALDI-TOF (m/z) (% relative intensity, ion): [M]^+^ calculated: 1546.90, observed: 1536.07

**Synthesis of 3c**

**
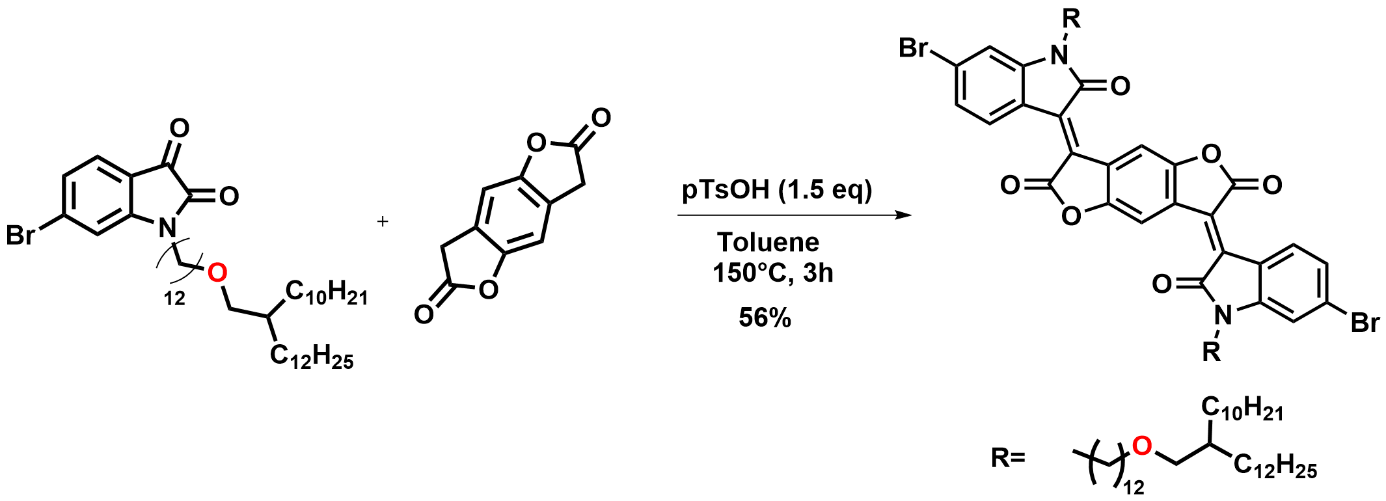
**

In a 250 mL round bottom flask **2c** (2 g, 2.67 mmol, 1.0 eq), benzo[1,2-b:4,5-b’]difuran-2,6(3H,7H)-dione , (0.25 g, 1.33 mmol, 0.5 eq), and p-toluenesulfonic acid (0.76 g, 4.00 mmol, 1.5 eq) were charged and the flask subjected to three evacuation/refill cycles with argon. Then the solids were dissolved in 80 mL of anhydrous toluene and the flask was equipped with a Dean-Stark apparatus. The mixture was refluxed under vigorous stirring for 3 h at 150 °C, in this time the color changed from bright orange to black. After this time the crude mixture was evaporated to dryness, the residue dissolved in chloroform and the organic phases washed three times with water (3x 50 mL) and brine (3x 50 mL). The organic layer was removed under vacuum and the residue was purified by column chromatography using petrol ether: dichloromethane (1:1) to yield a powdery black/green solid (1.12 g, 50 %).

^1^H NMR (600.1 MHz, CDCl_3_) δ / ppm 8.95 (s, 1H, CH_BDF_), 8.78 (d, *^3^J_HH_* = 8.73 Hz, 1 H, -CH_(4)_), 7.09 (d *^3^J_HH_* = 8.73 Hz, 1 H, CH_(5)_), 6.91 (s, 1 H, CH_(1)_), 3.70 (t, *^3^J_HH_* = 7.30 Hz, 2 H, N-CH_2_-), 3.62 (t, *^3^J_HH_* = 7.67 Hz, 2 H, N-CH_2_-), 3.29 (t, *^3^J_HH_* = 6.14 Hz, 2 H, -CH_2_-O-DT), 3.18 (d, *^3^J_HH_* = 5.96 Hz, 2 H, -O-CH_2_-CHR2), 1.80-1.1 (m, 56 H, -(CH_2_)_8_-/DT_internal_), 0.81 (t, *^3^J_HH_* = 7.11 Hz, 6 H, DT_terminal_).

^13^C{^1^H} NMR (150.9 MHz, CDCl3) δ / ppm 167.0 (C=O_amide_), 151.3 (C=O_BDF_), 146.8 (C_2isatin)_, 135.5 (C=C_isatin_), 131,5 (C_4isatin_),129.1 (C_Br_) 126.8 (C=C_BDF_), 126.4 (C_qBDF_), 125.6 (C_5isatin_), 119.7 (C_3isatin_), 111.8(CH_BDF_) 111.0 (C_1isatin_), 74.4 (CH_2_O-DT), 70.1 (-O-CH_2_-CHR2), 38.3 (O-CH_2_-CHR2), 32.1-22.7 (DT_internal_), 14.2 (DT_terminal_).

MALDI-TOF (m/z) (% relative intensity, ion): [M]^+^ calculated: 1648.12, observed: 1648.05.

**Polymerizations**

**
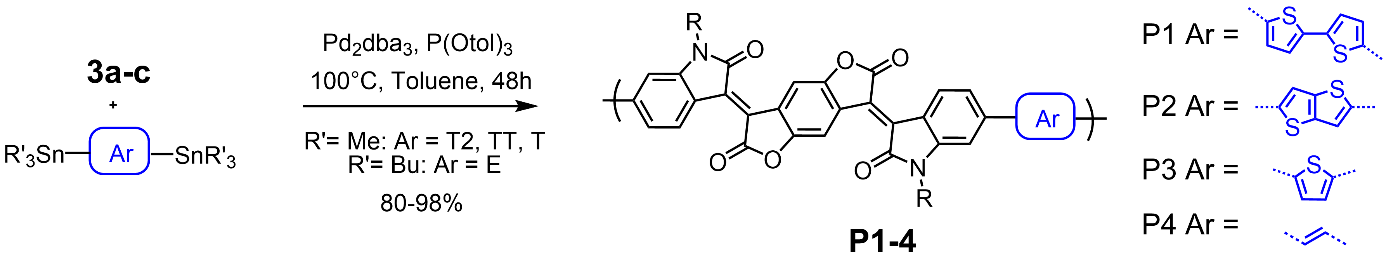
**

General procedure of polymerization: In a high pressure septum vial, monomers **3a-c** were added in a 1:1 molar ratio along with the stannylated comonomers. To the vial Pd_2_dba_3_·CHCl_3_ (1% mol with respect to monomer) and P(*o*-tol)_3_ (5 mol % respective to monomer) were added. The vials were subjected to three vacuum/argon cycles and were further purged with argon for 15 min after the last cycle. Finally, sodium and dried and degassed toluene was added until the monomer concentration was 0.01 M. The vials were then placed in a heating plate at 100 °C for 48 h. After this time, the crude reactions were allowed to reach room temperature, and then the mixtures were precipitated in 300 mL of methanol under vigorous stirring. The precipitate was collected using filter paper. The filter was placed in a Soxhlet apparatus and was subjected to Soxhlet extraction for 4 h with acetone, 12 h with ethyl acetate, 4 h with petrol ether. The Soxhlet was charged with CHCl_3_ and the polymer collected. Finally, the solvent was concentrated under reduced pressure and the polymer re-precipitated in 300 mL of methanol to yield black solids in 87-97 % yield.

**2 Additional Figures**


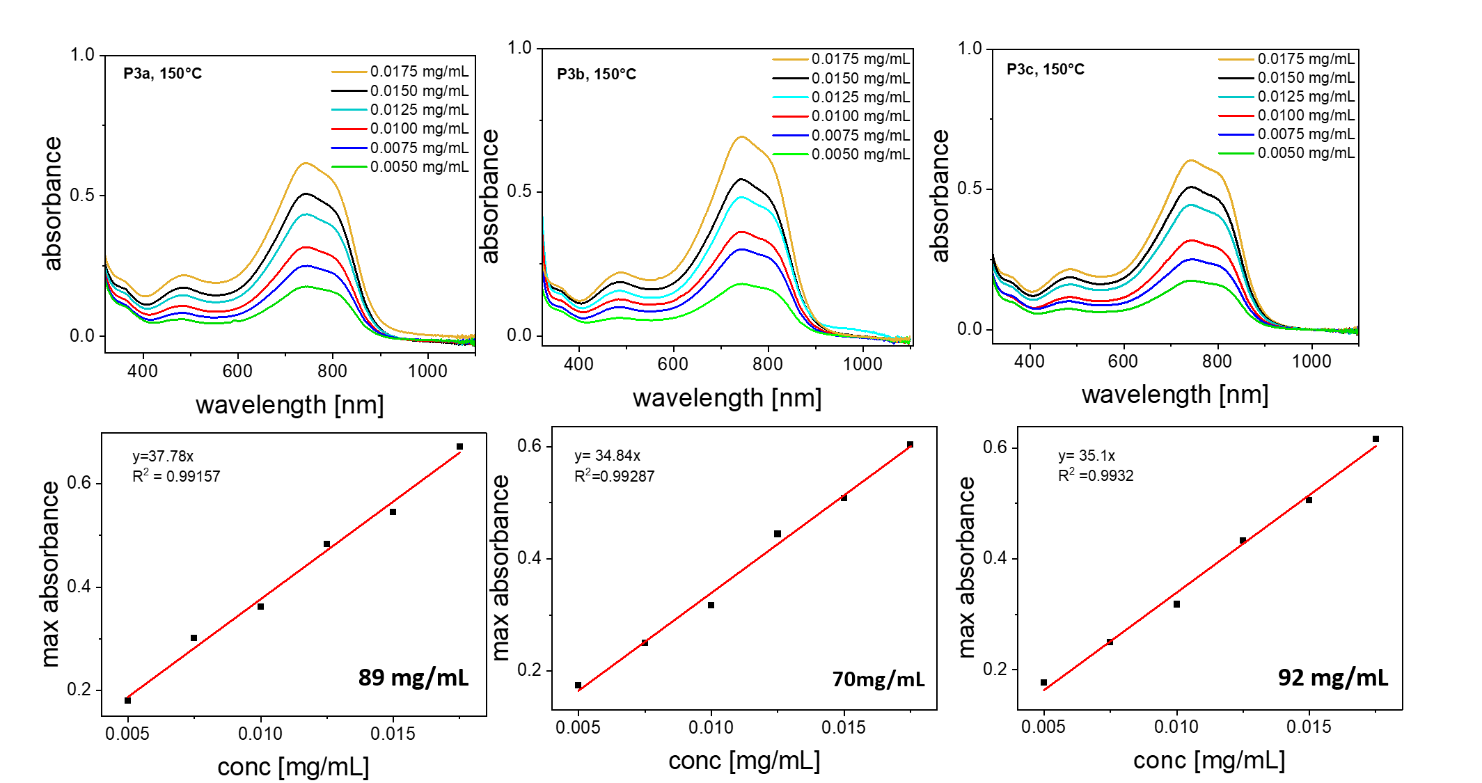


**Figure S1.** Solubility saturation determination for P3a-c.


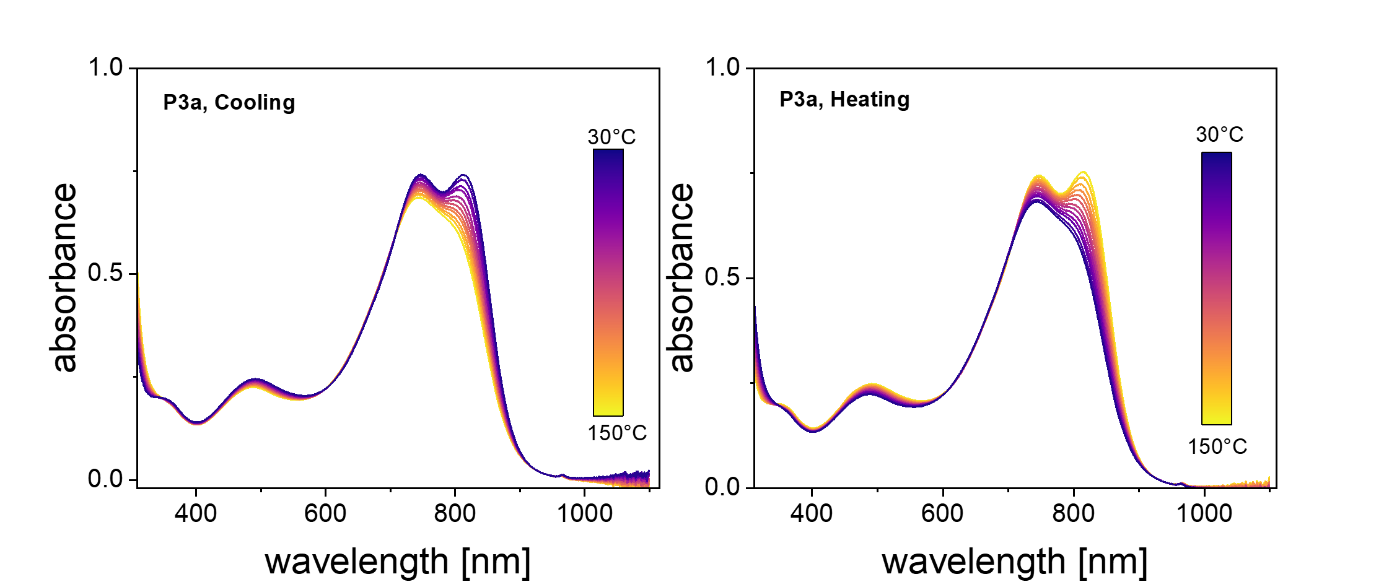

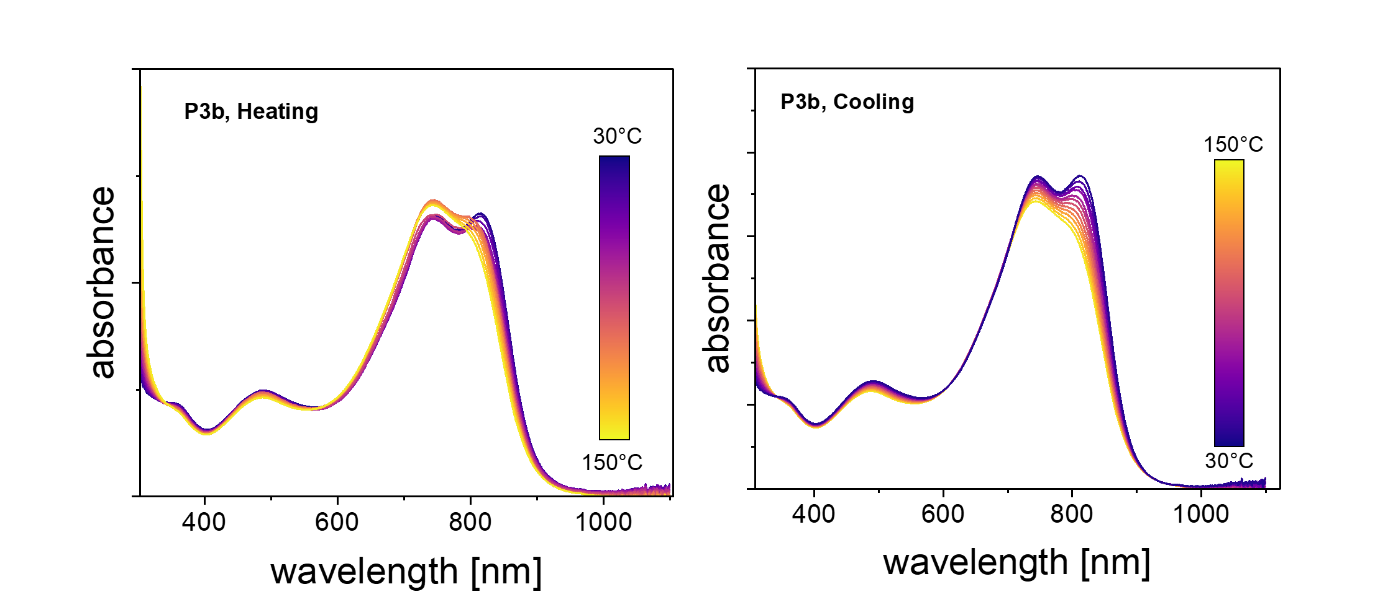

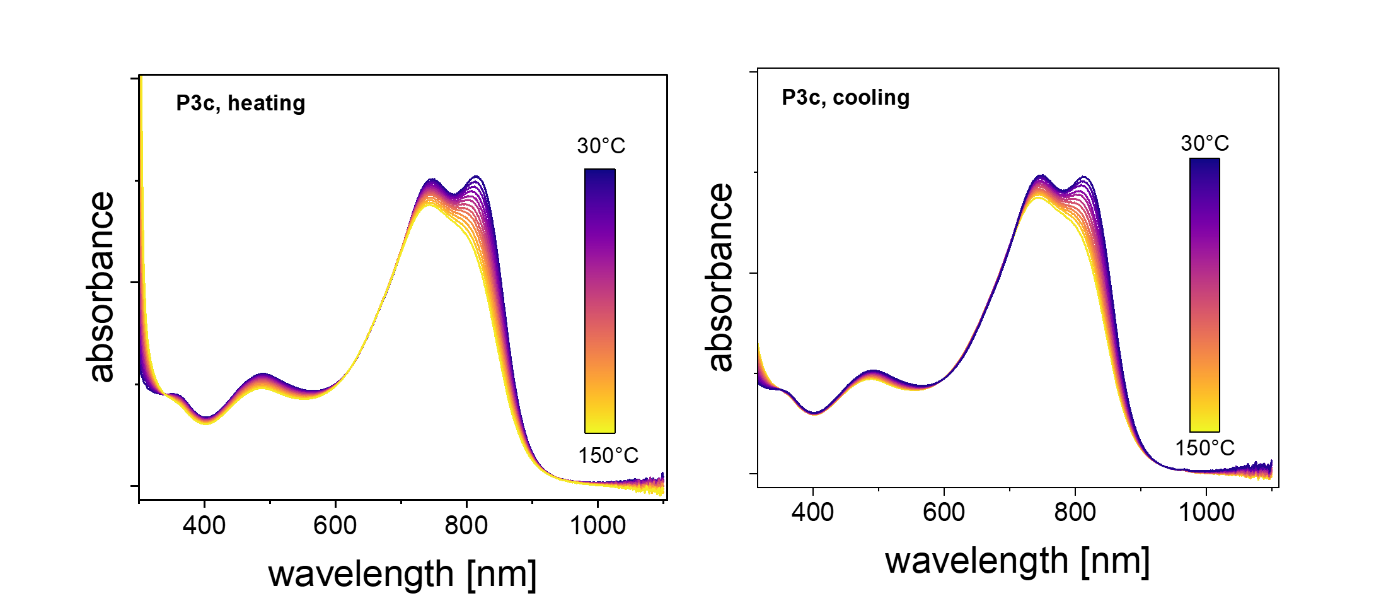


**Figure S2.** Variable temperature UV-vis spectra of P3a, P3b and P3c in *o-*DCB.


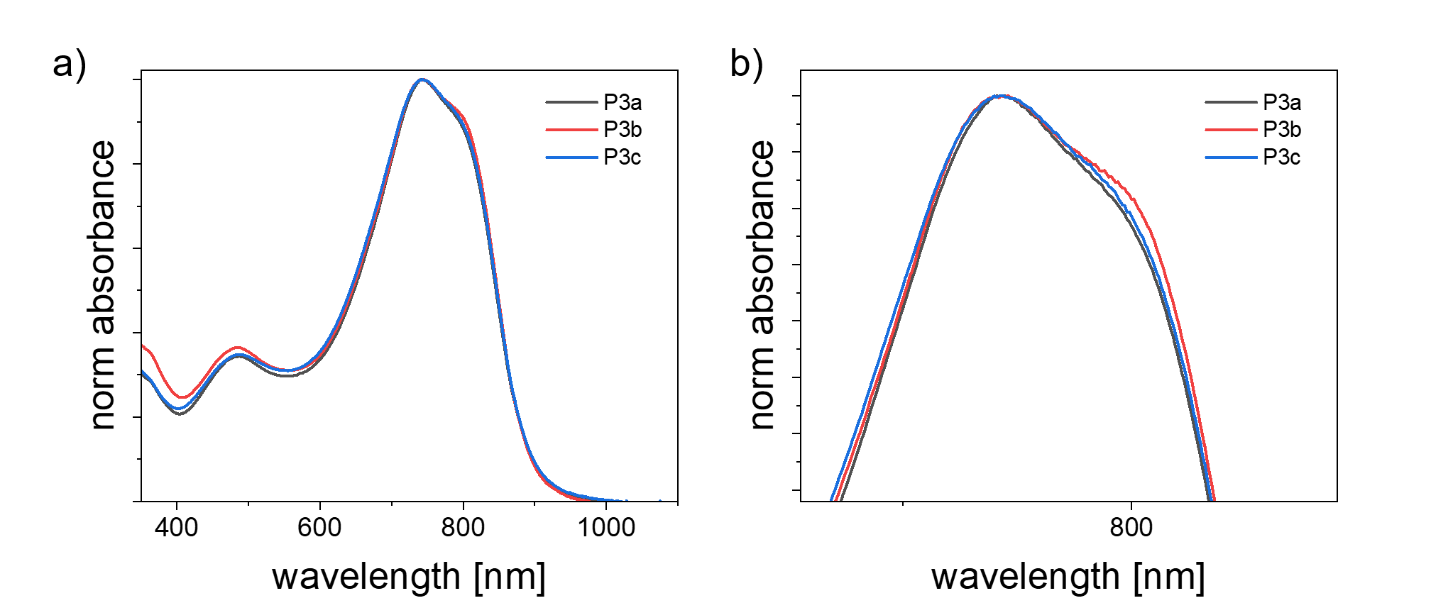


**Figure S3.** a) UV-vis spectra of P3a-c in *o-*DCB at 150°C. b) Enlarged region of the shoulder at 820 nm.


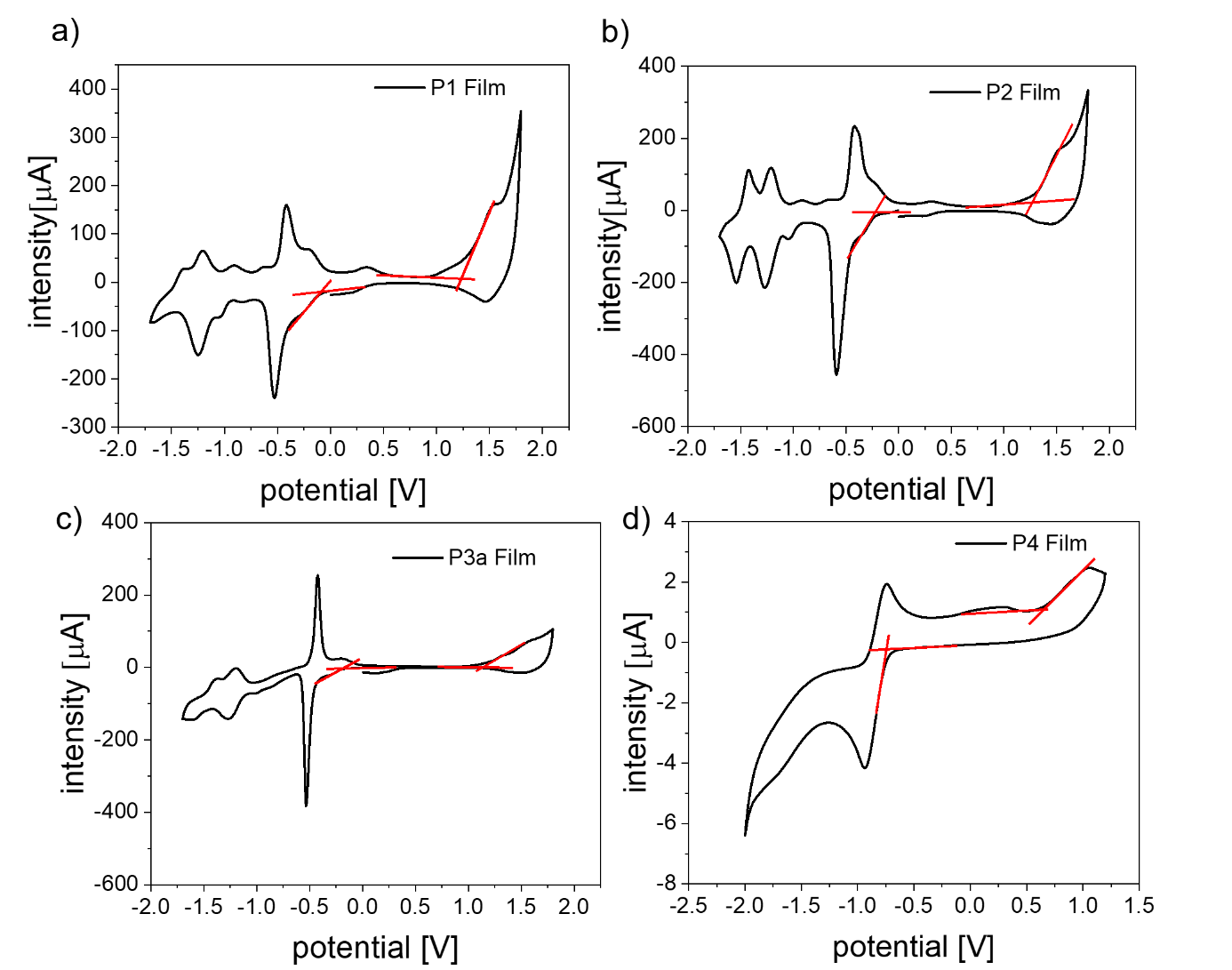


**Figure S4.** Cyclic voltammetry of films of a) P1, b) P2, c) P3a and d) P4.


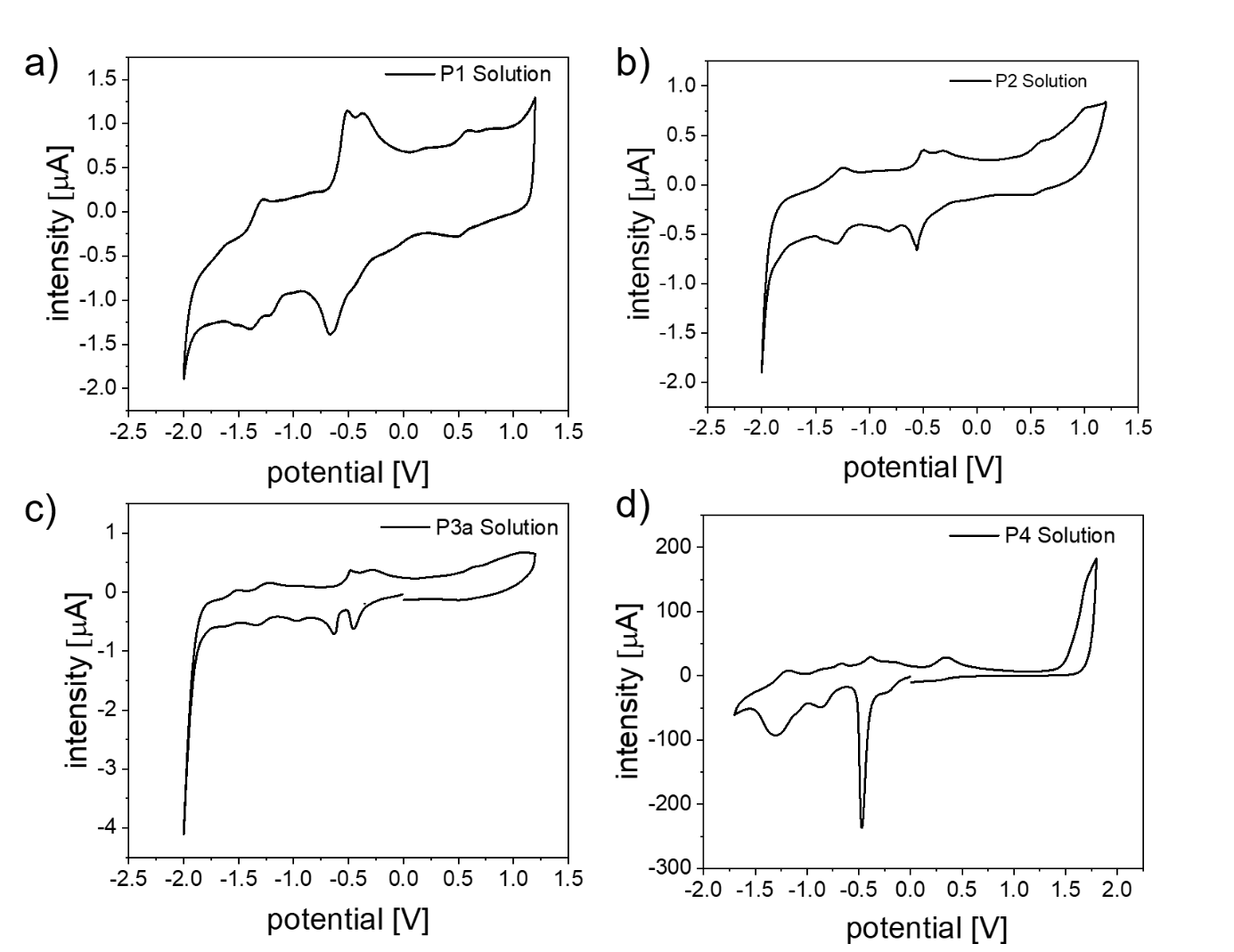


**Figure S5.** Cyclic voltammetry of solutions of a) P1, b) P2, c) P3a and d) P4.


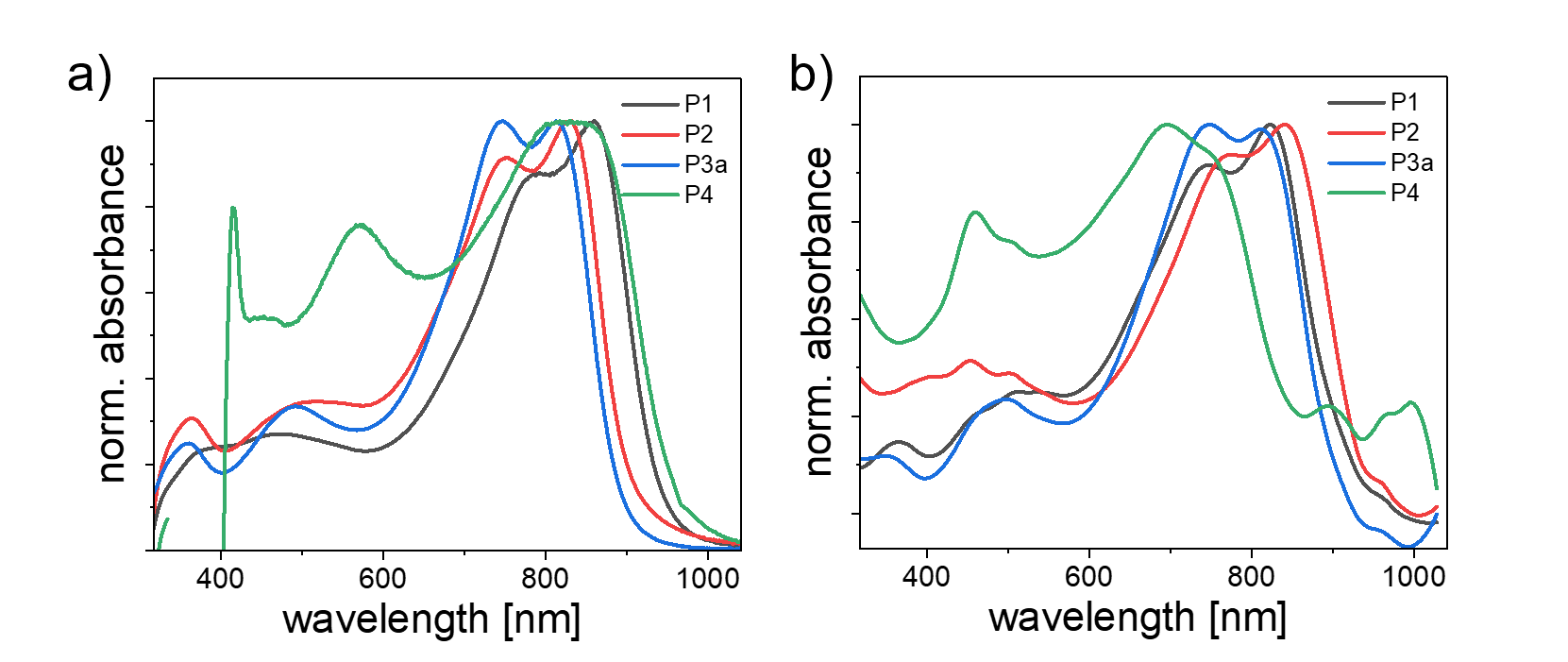


**Figure S6.** UV-vis spectra of P1, P2, P3a and P4 in a) *o-*DCB solution at 25°C and b) in film.


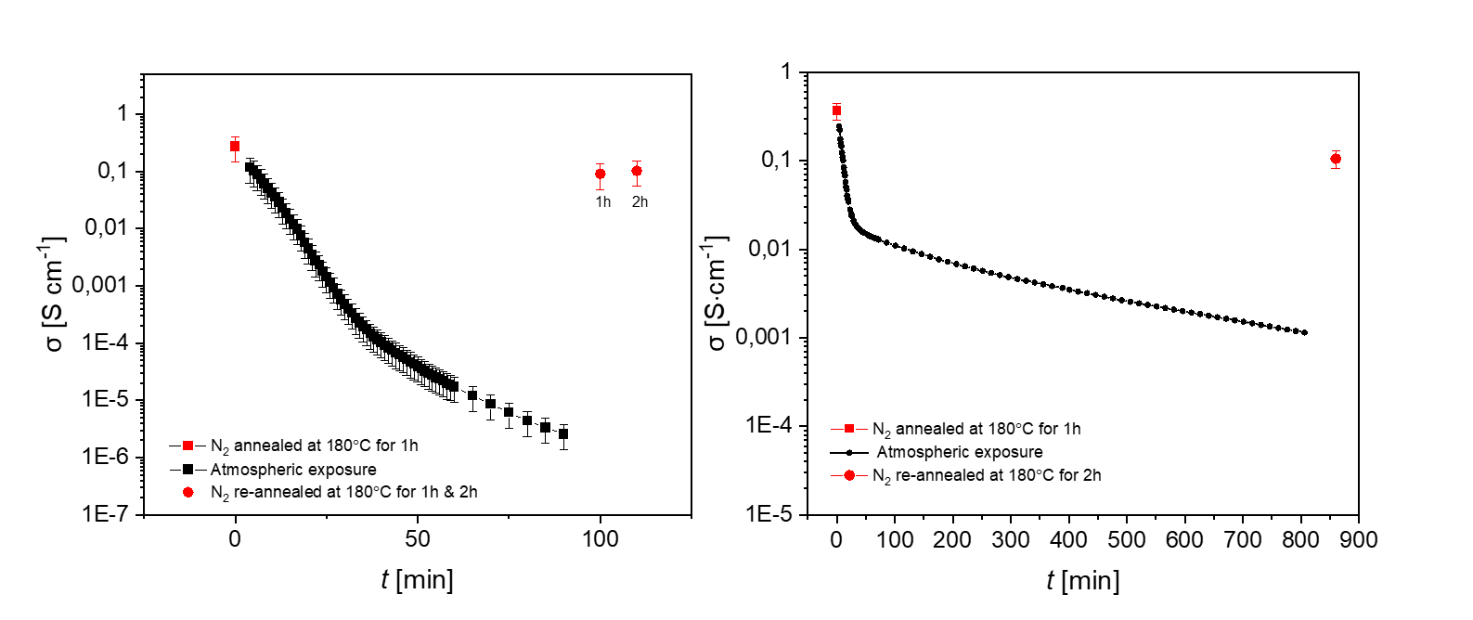


**Figure S7.** Stability tests of P3a thin films spin-cast from *o*-DCB (left) and toluene (right)

doped with 50 MR% N-DMBI.


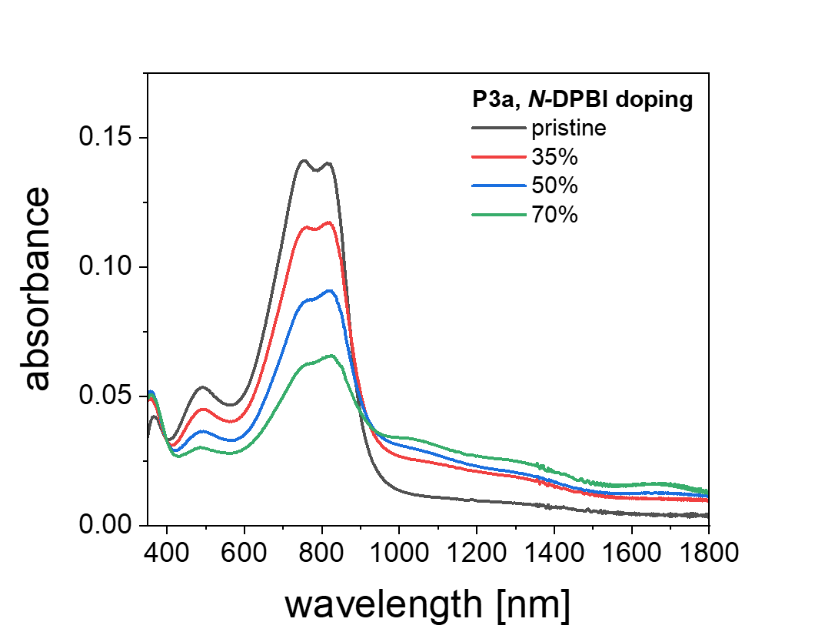


**Figure S8.** UV-vis spectra of P3a at different *N-*DPBI doping concentrations.


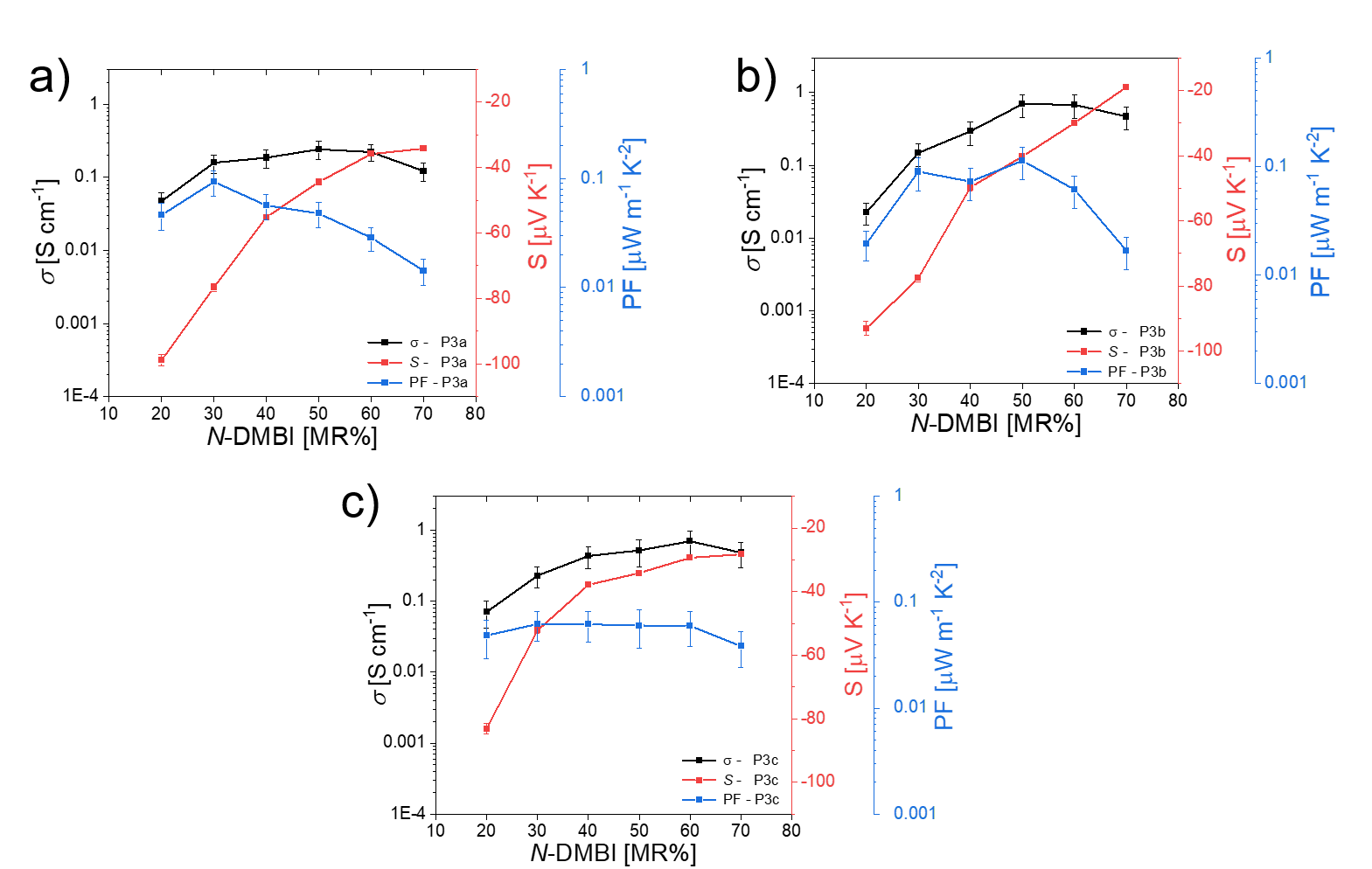


**Figure S9.** Thermoelectrical characterization of a) P3a, b) P3b and c) P3c.


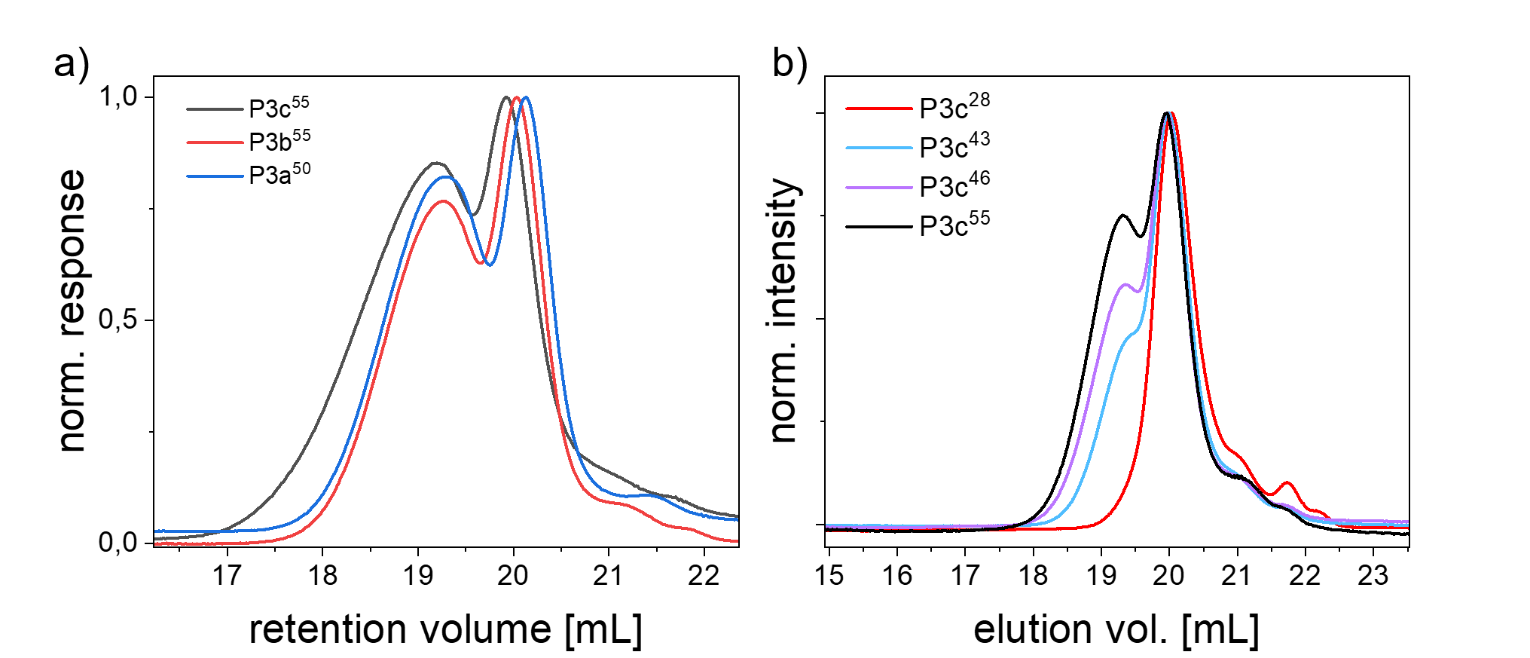


**Figure S10.** a) SEC traces in TCB at 150 °C for P3a-c. b) SEC traces in TCB at 150 °C for the different Mn P3c samples.

**3 NMR Spectra**


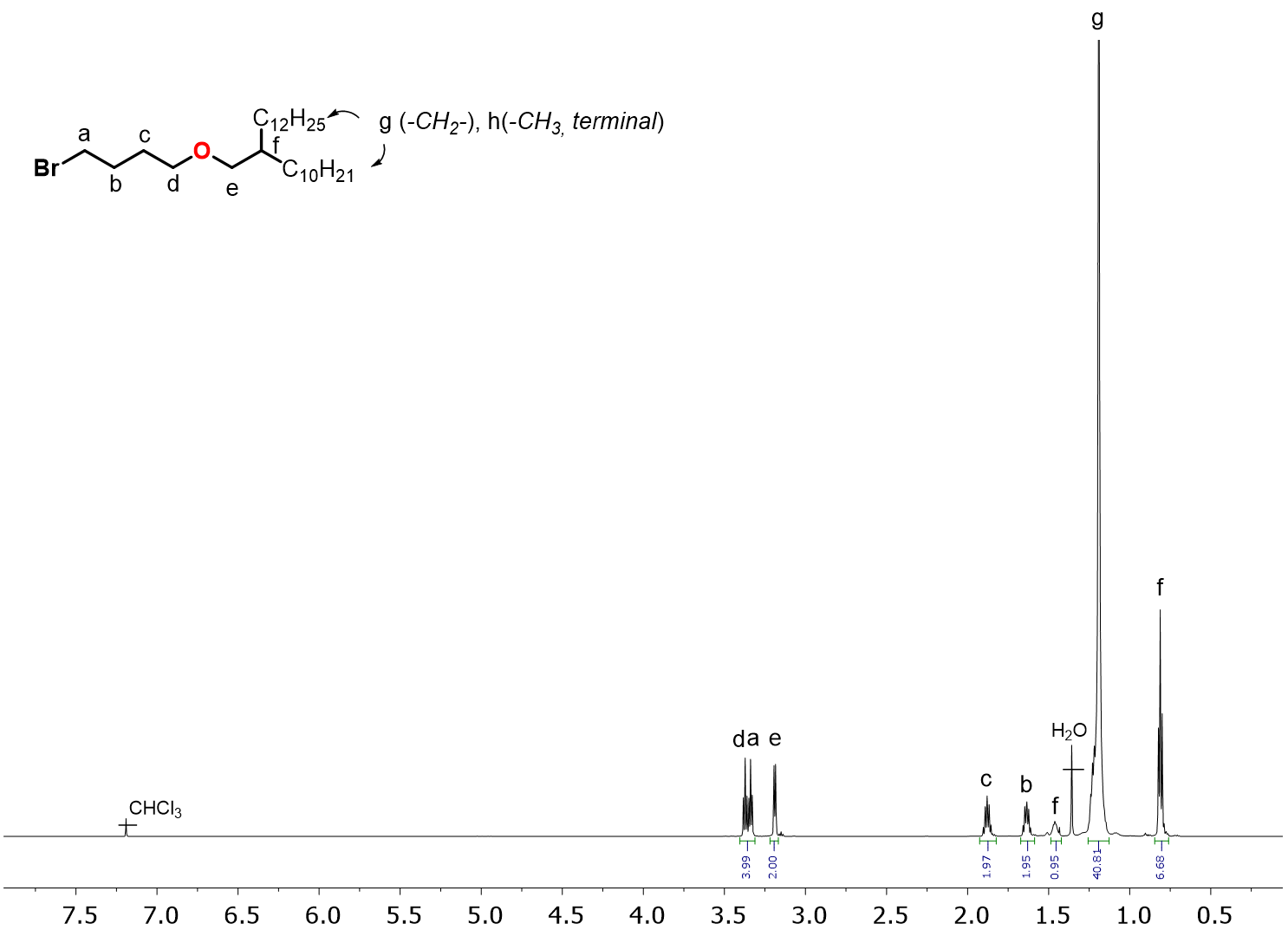


**Figure S11.** ^1^H-NMR spectrum of (**1a**) in CDCl_3_ at 298K.


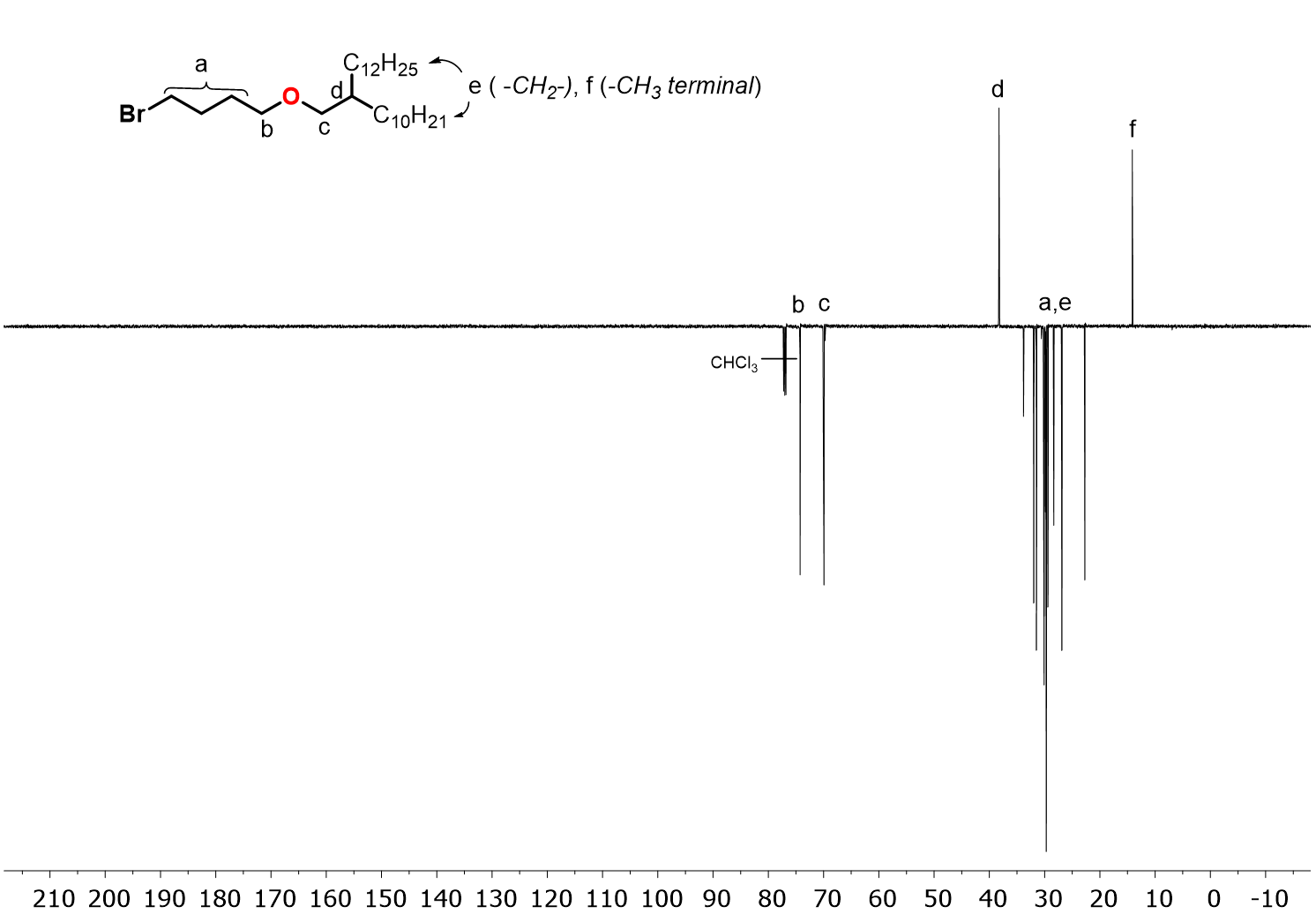


**Figure S12.** ^13^C{^1^H}-APT spectrum of (**1a**) in CDCl3 at 298K.


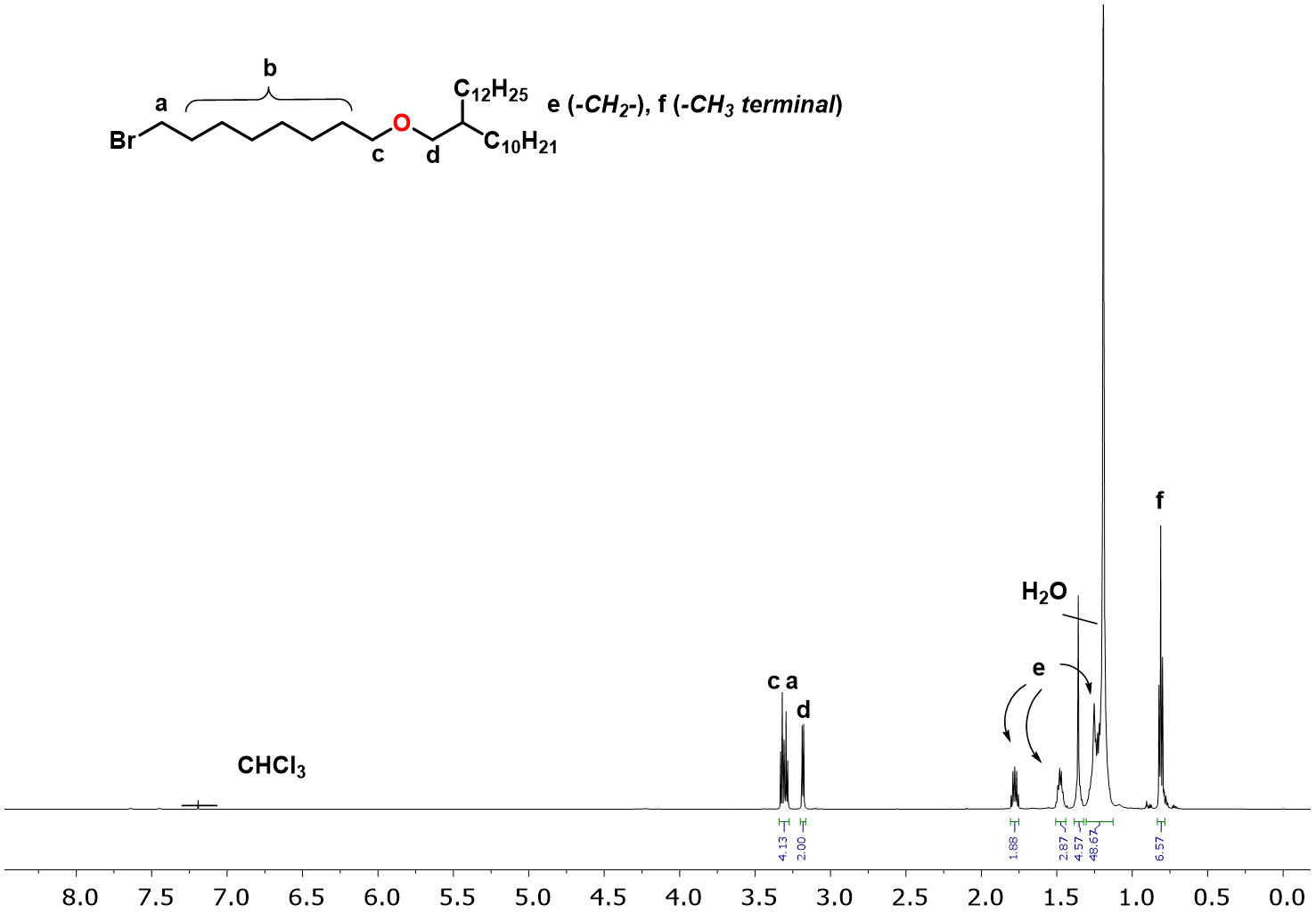


**Figure S13.** ^1^H-NMR spectrum of (**2a**) in CDCl_3_ at 298K.


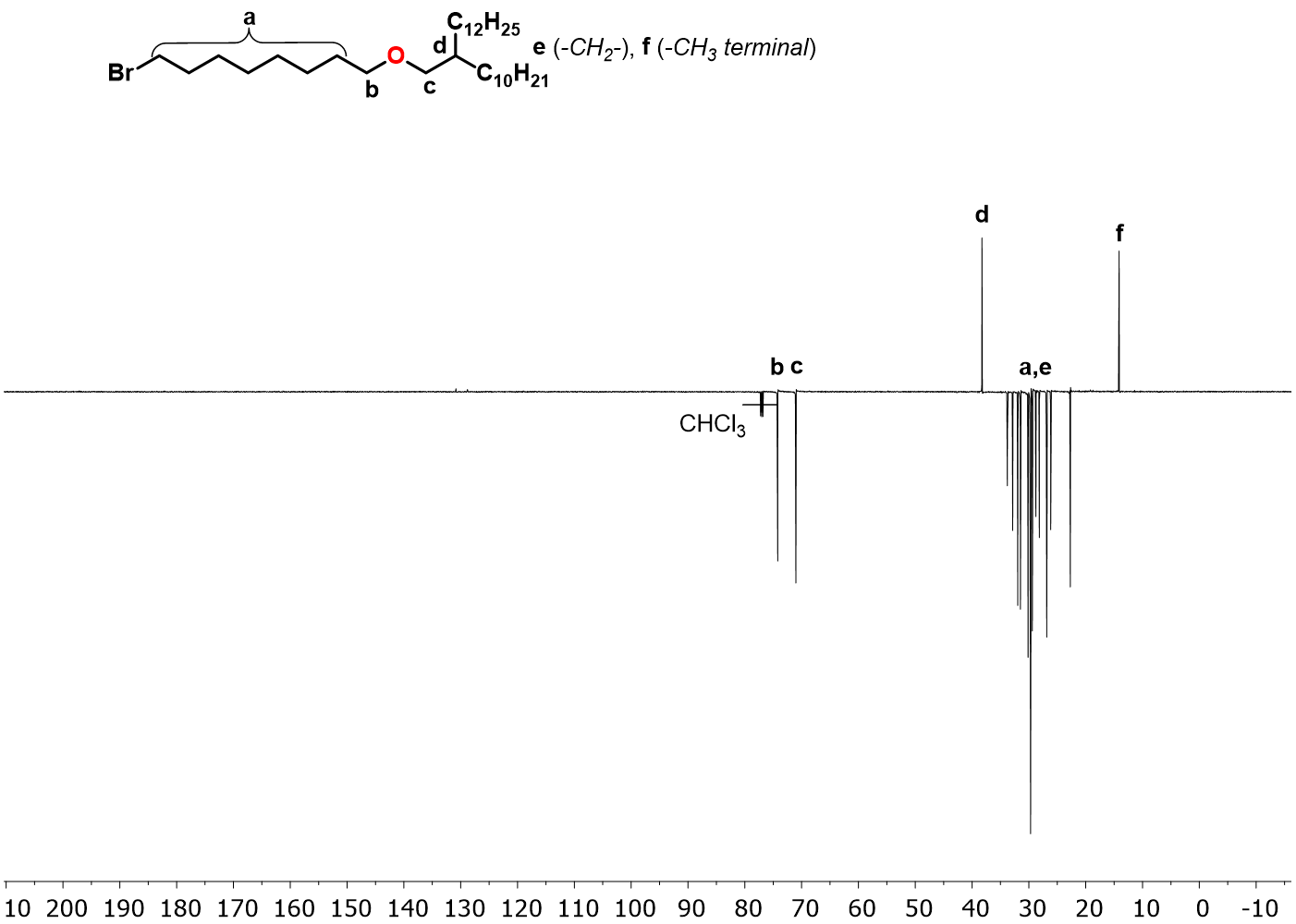


**Figure S14.** ^13^C{^1^H}-APT spectrum of (**2a**) in CDCl3 at 298K.


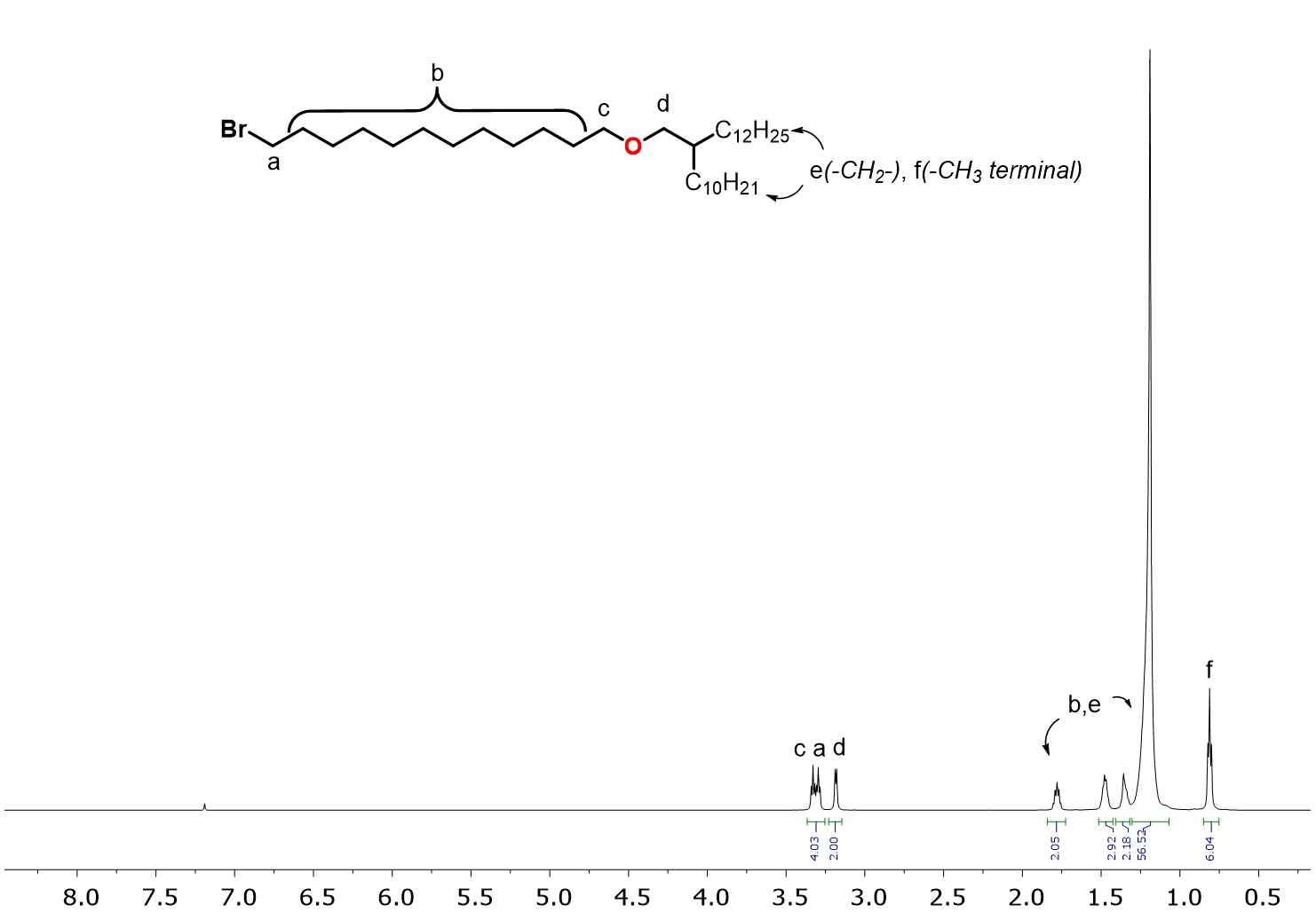


**Figure S15.** ^1^H-NMR spectrum of (**3a**) in CDCl_3_ at 298K.


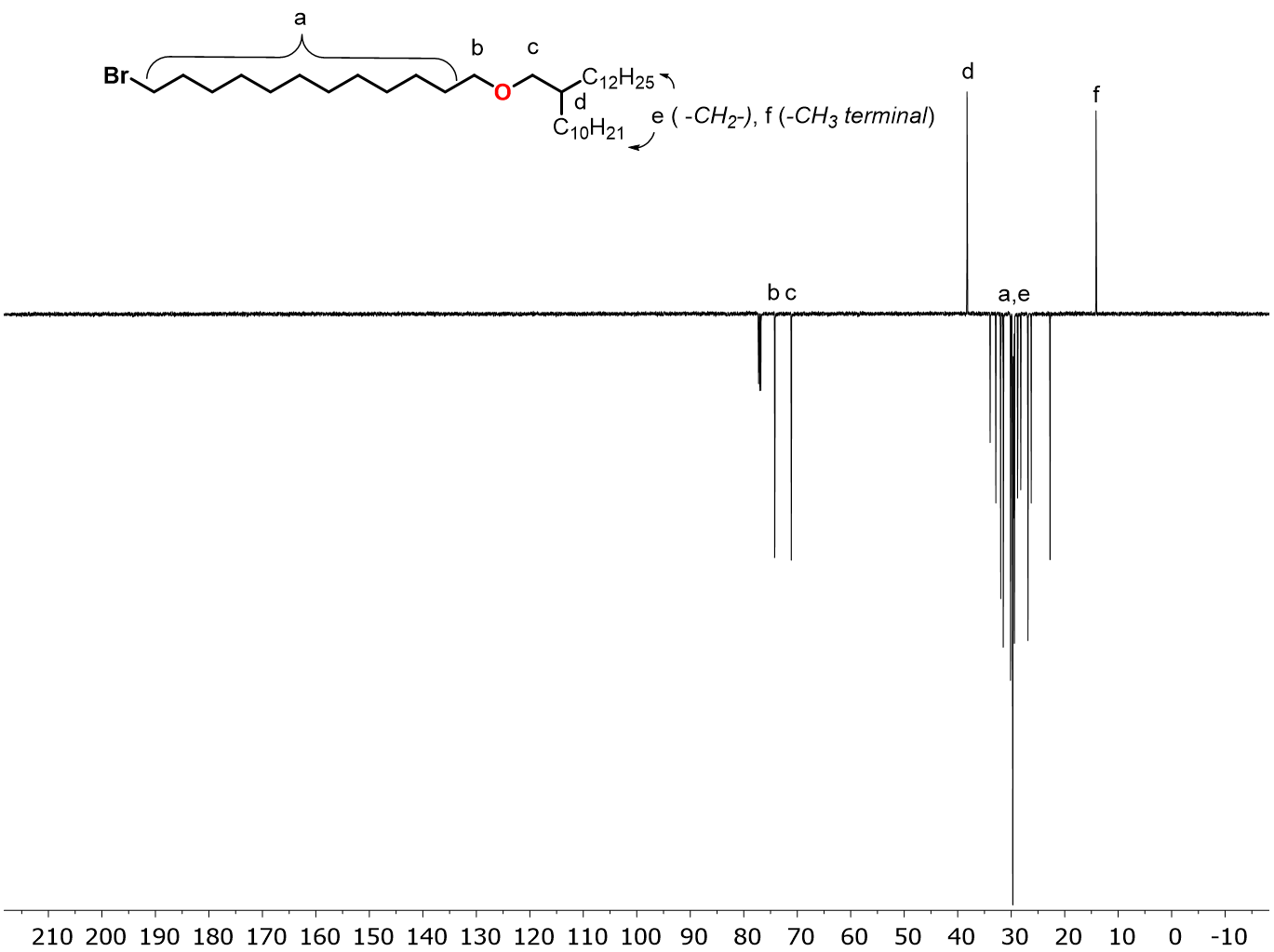


**Figure S16.** ^13^C{^1^H}-APT spectrum of (**3a**) in CDCl3 at 298K.


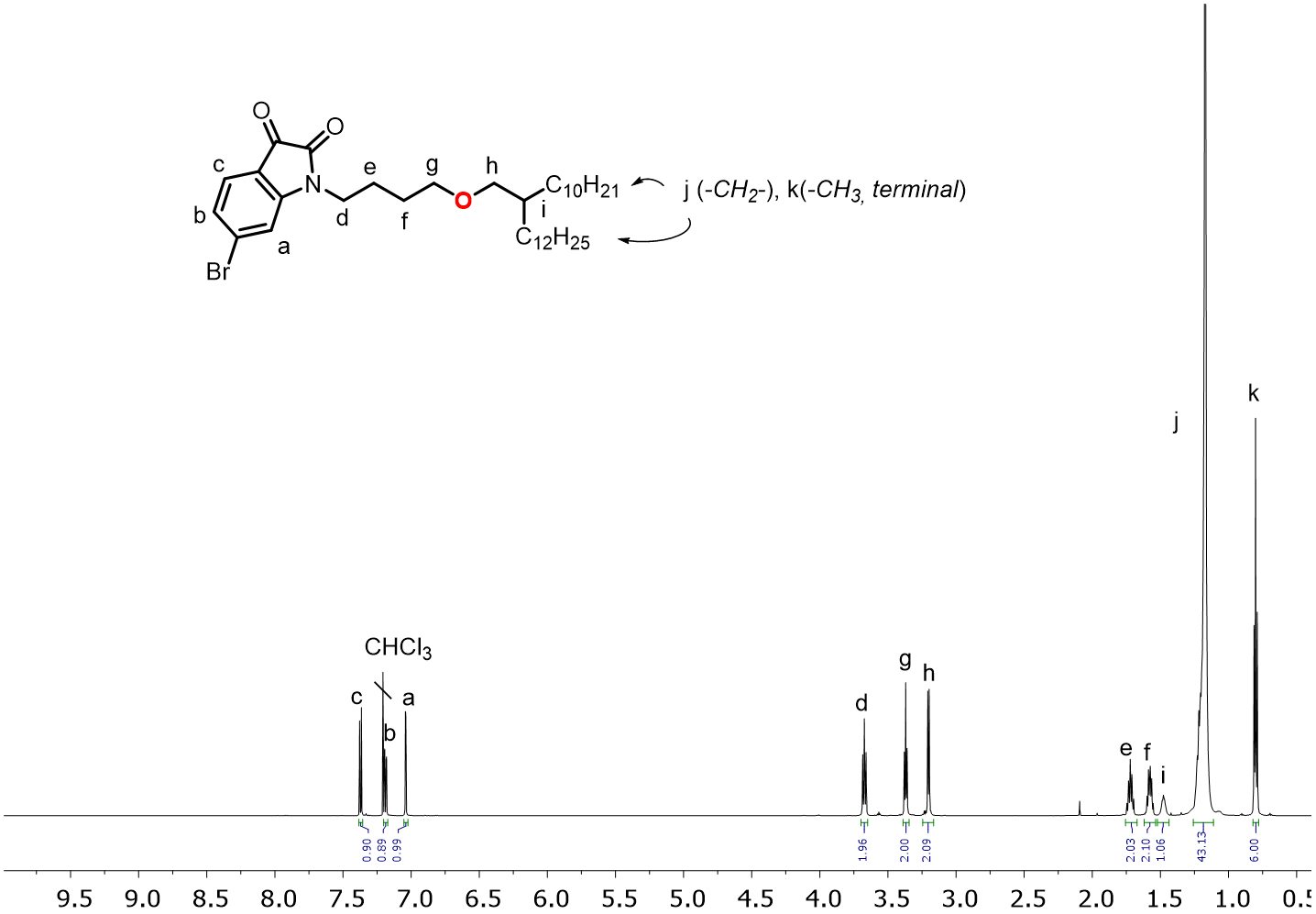


**Figure S17.** ^1^H-NMR spectrum of (**2a**) in CDCl_3_ at 298K.


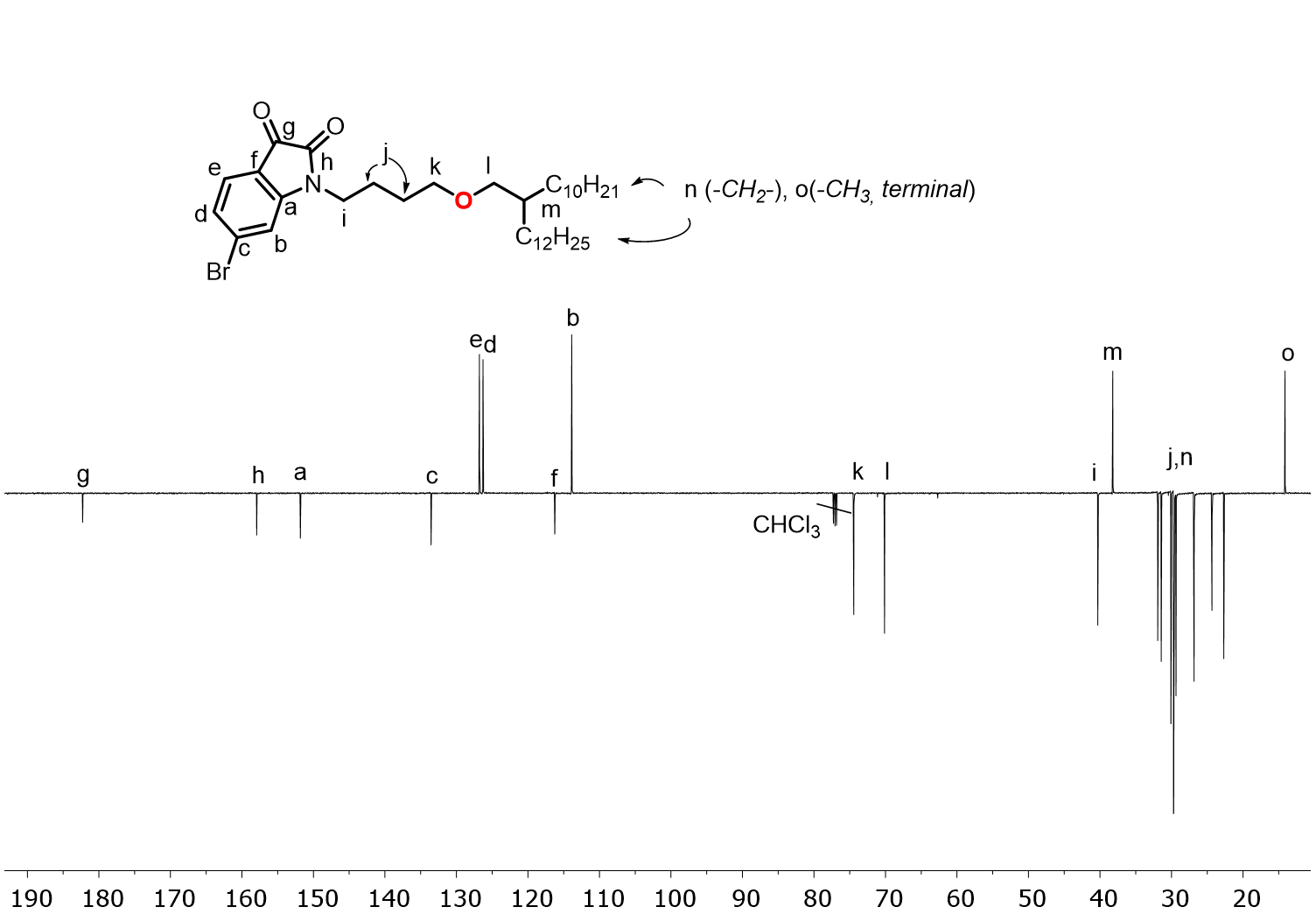


**Figure S18.** ^13^C{^1^H}-APT spectrum of (**2a**) in CDCl3 at 298K.


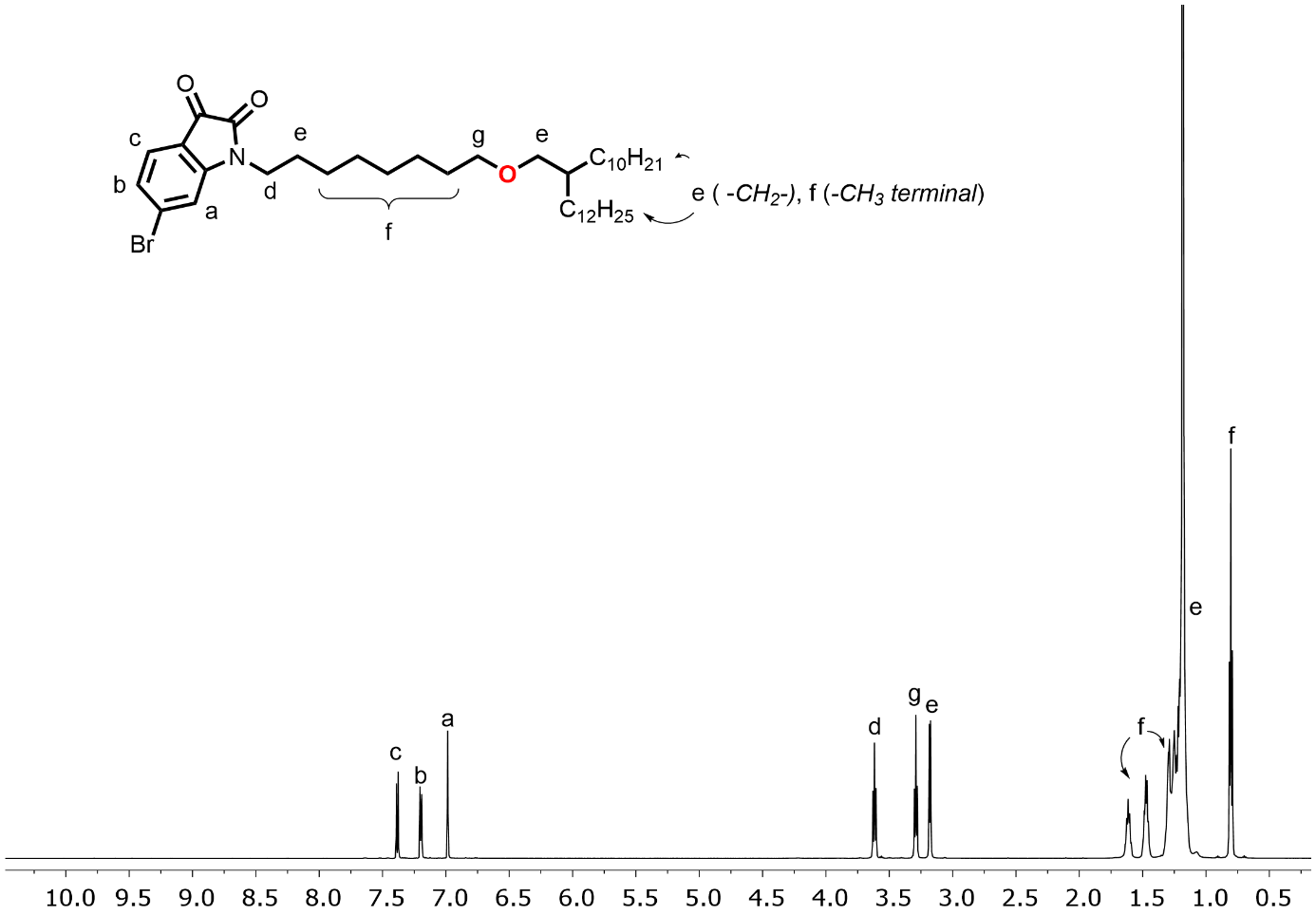


**Figure S19.** ^1^H-NMR spectrum of (**2b**) in CDCl_3_ at 298K.


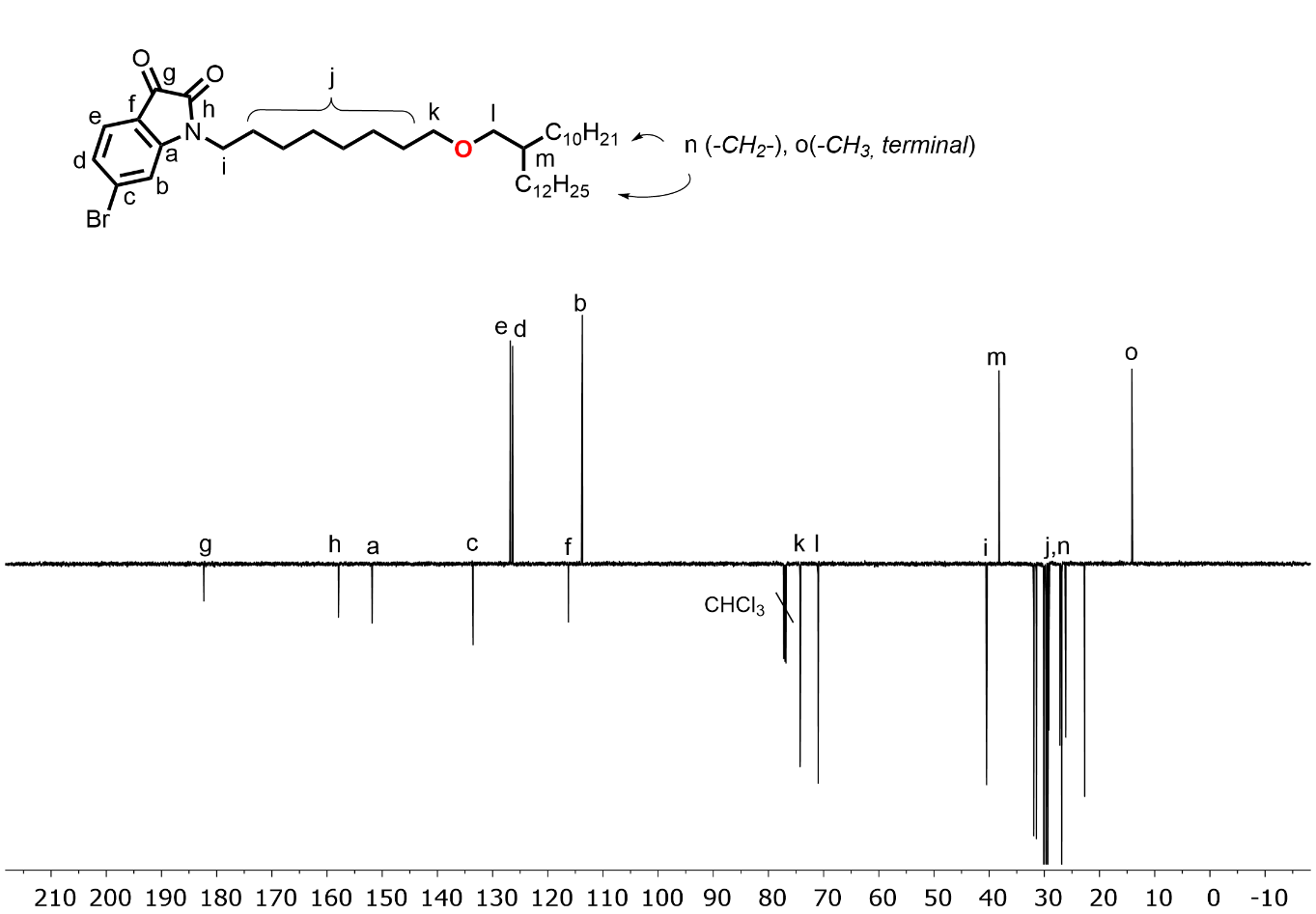


**Figure S20.** ^13^C{^1^H}-APT spectrum of (**2b**) in CDCl3 at 298K.

**
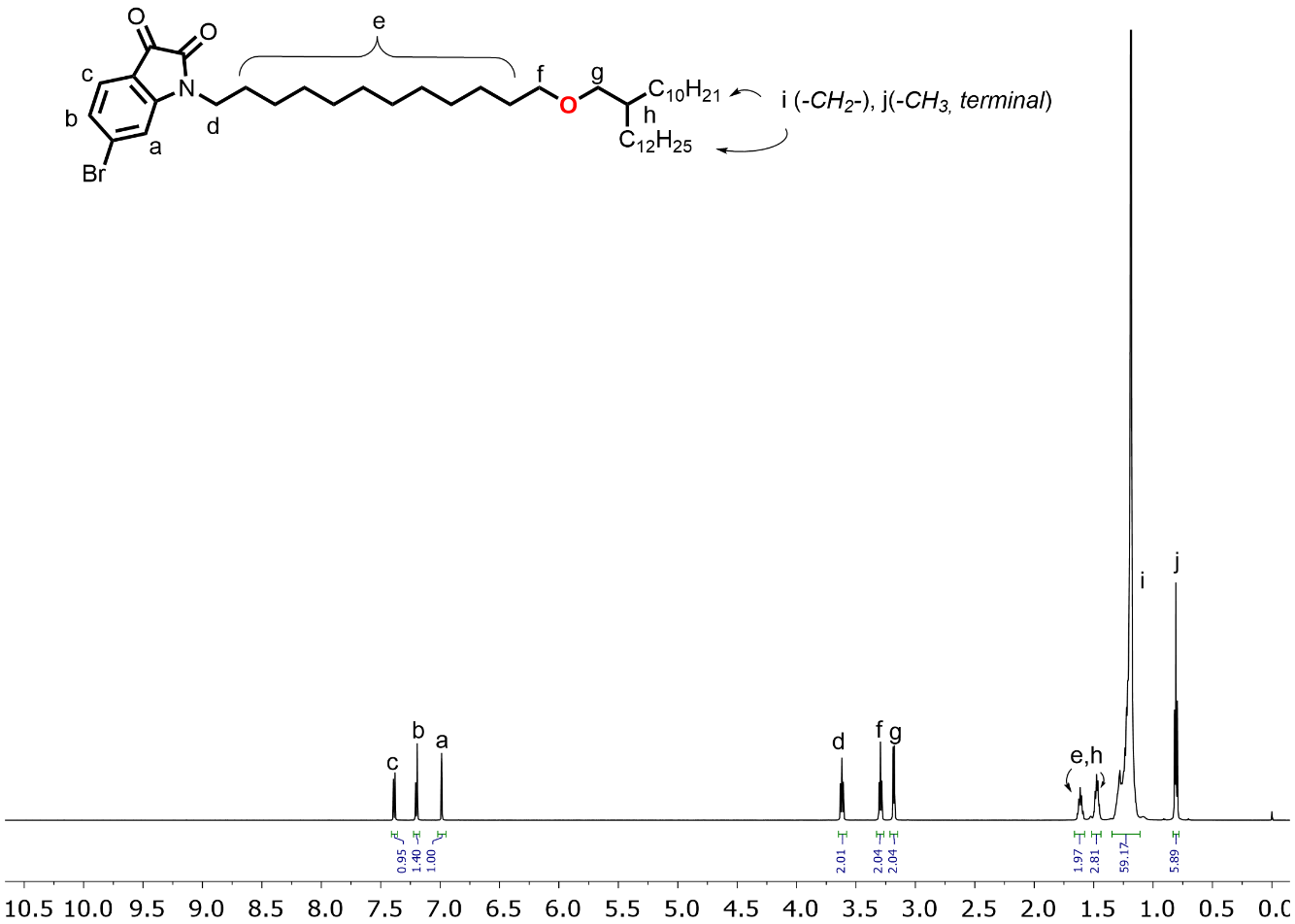
**

**Figure S21.** ^1^H-NMR spectrum of (**2c**) in CDCl_3_ at 298K.


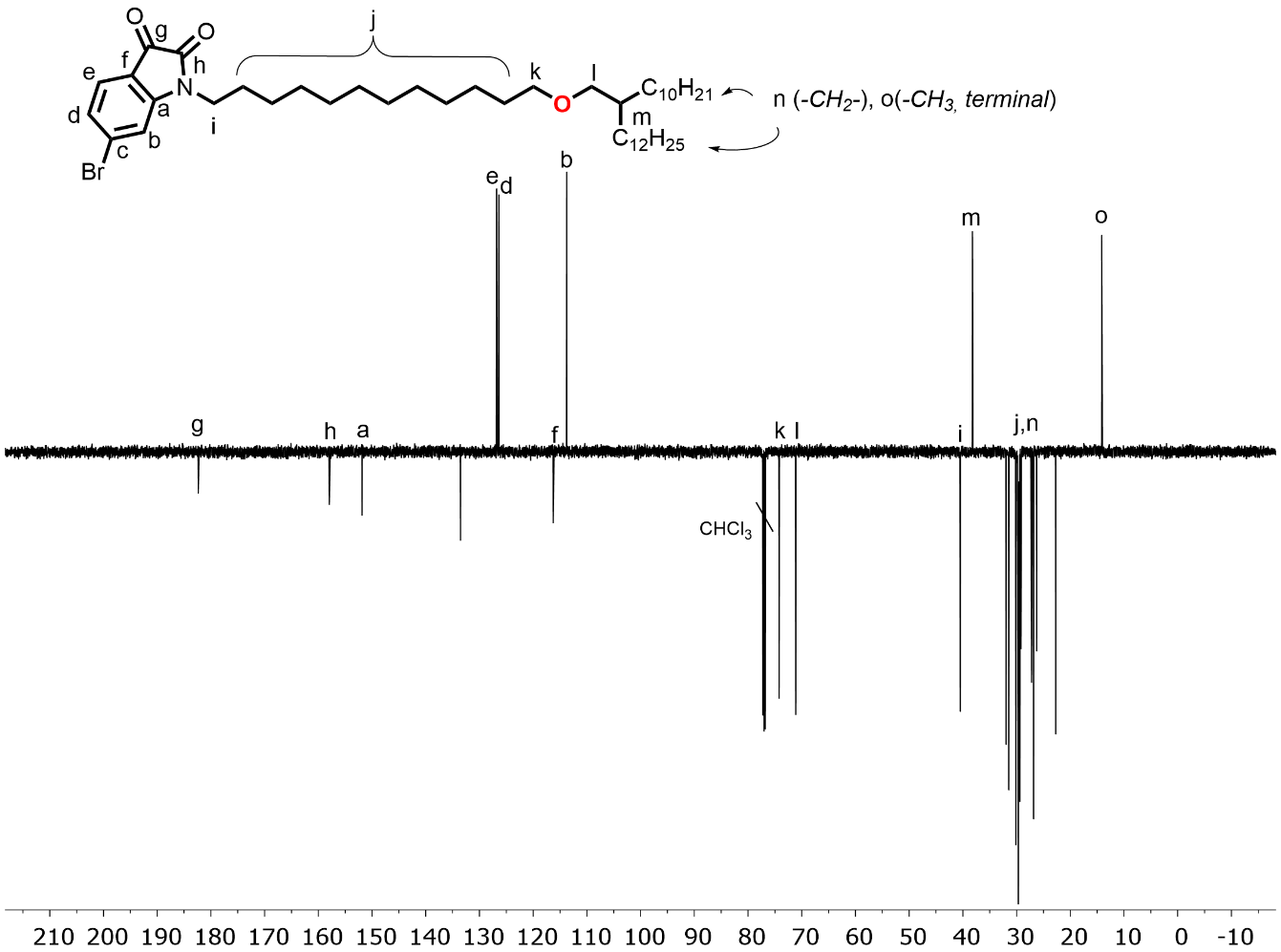


**Figure S22.** ^13^C{^1^H}-APT spectrum of (**2c**) in CDCl3 at 298K.


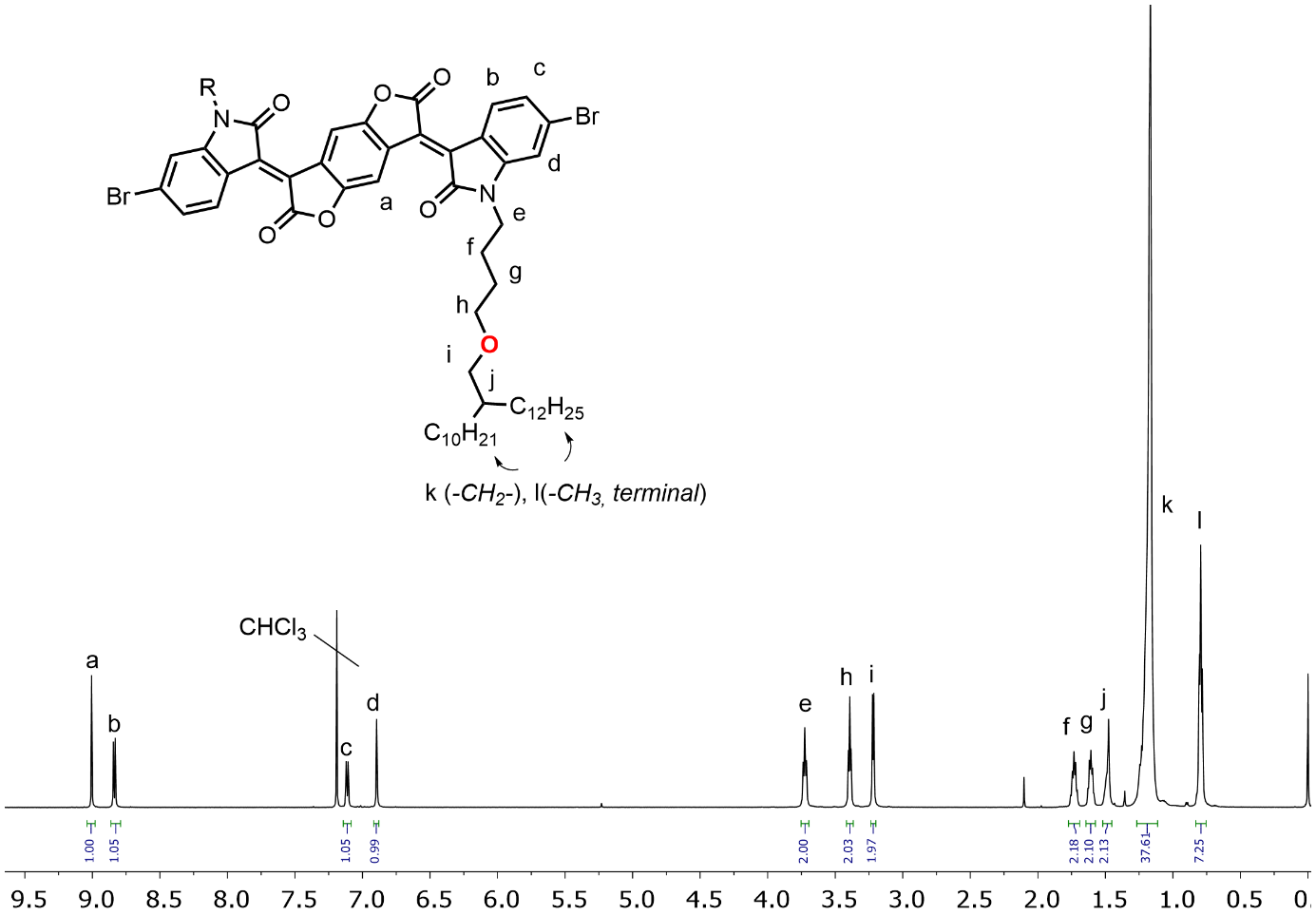


**Figure S23.** ^1^H-NMR spectrum of (**3a**) in CDCl_3_ at 298K.


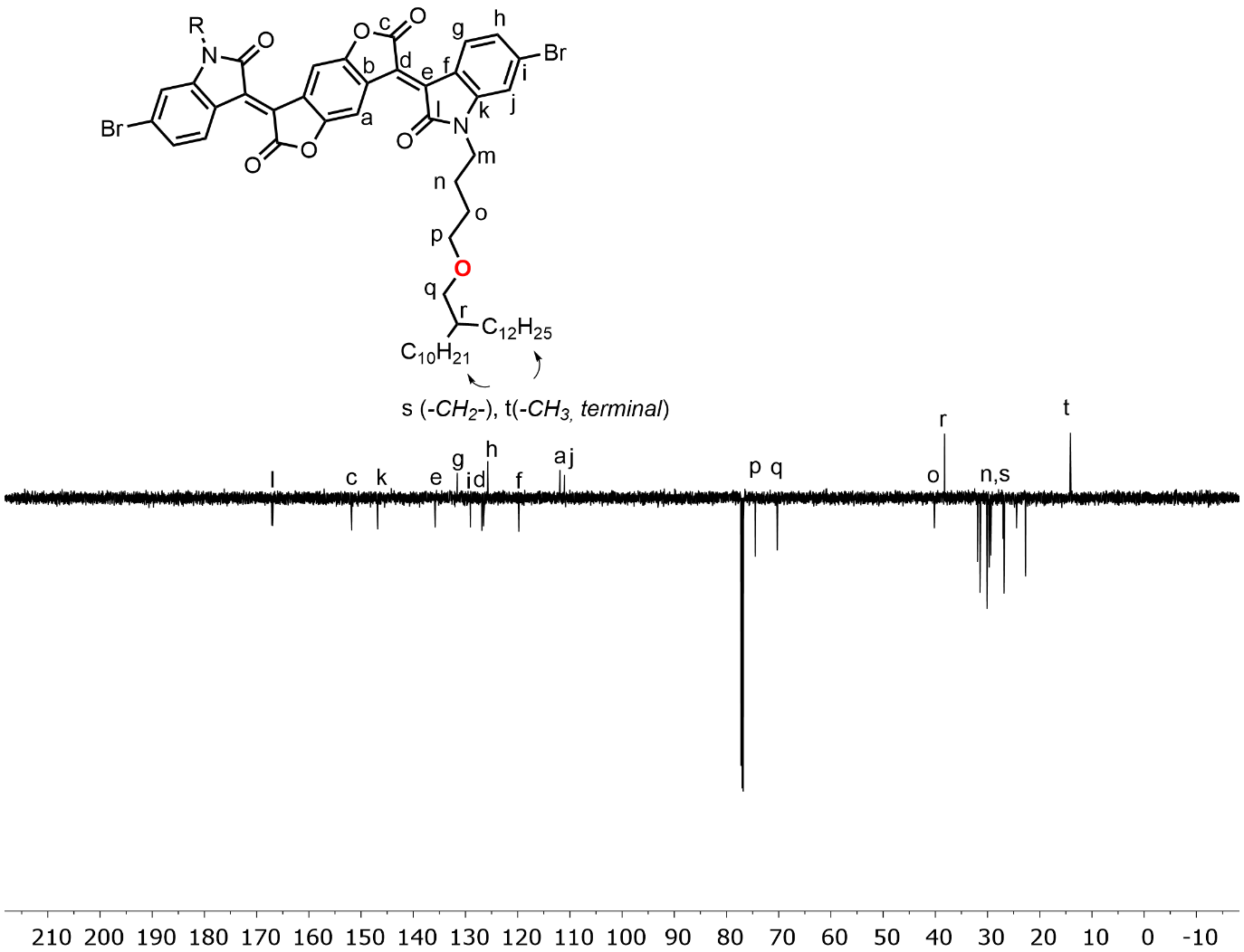


**Figure S24.** ^13^C{^1^H}-APT spectrum of (**3a**) in CDCl3 at 298K.


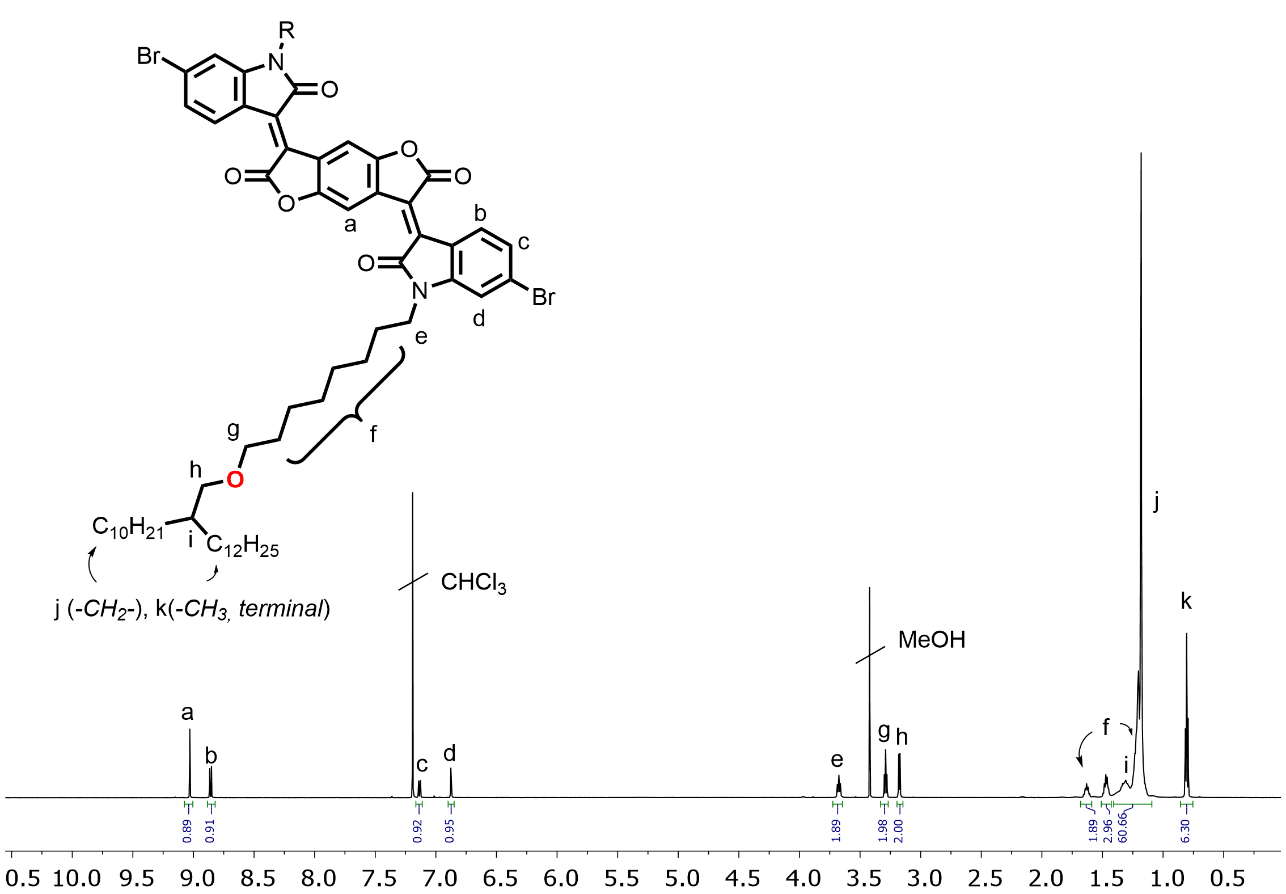


**Figure S25.** ^1^H-NMR spectrum of (**3b**) in CDCl_3_ at 298K.


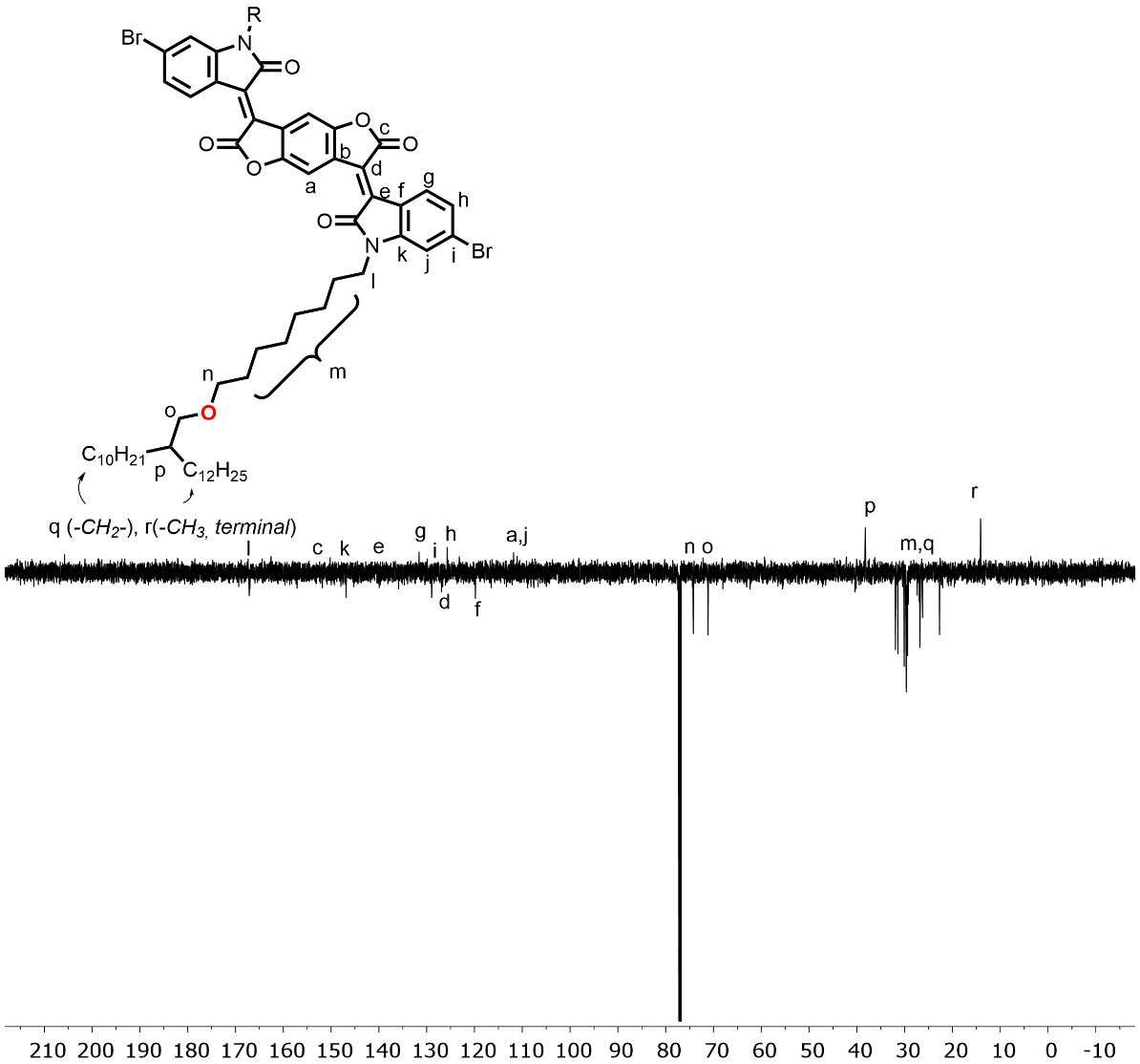


**Figure S26.** ^13^C{^1^H}-APT spectrum of (**3a**) in CDCl3 at 298K.


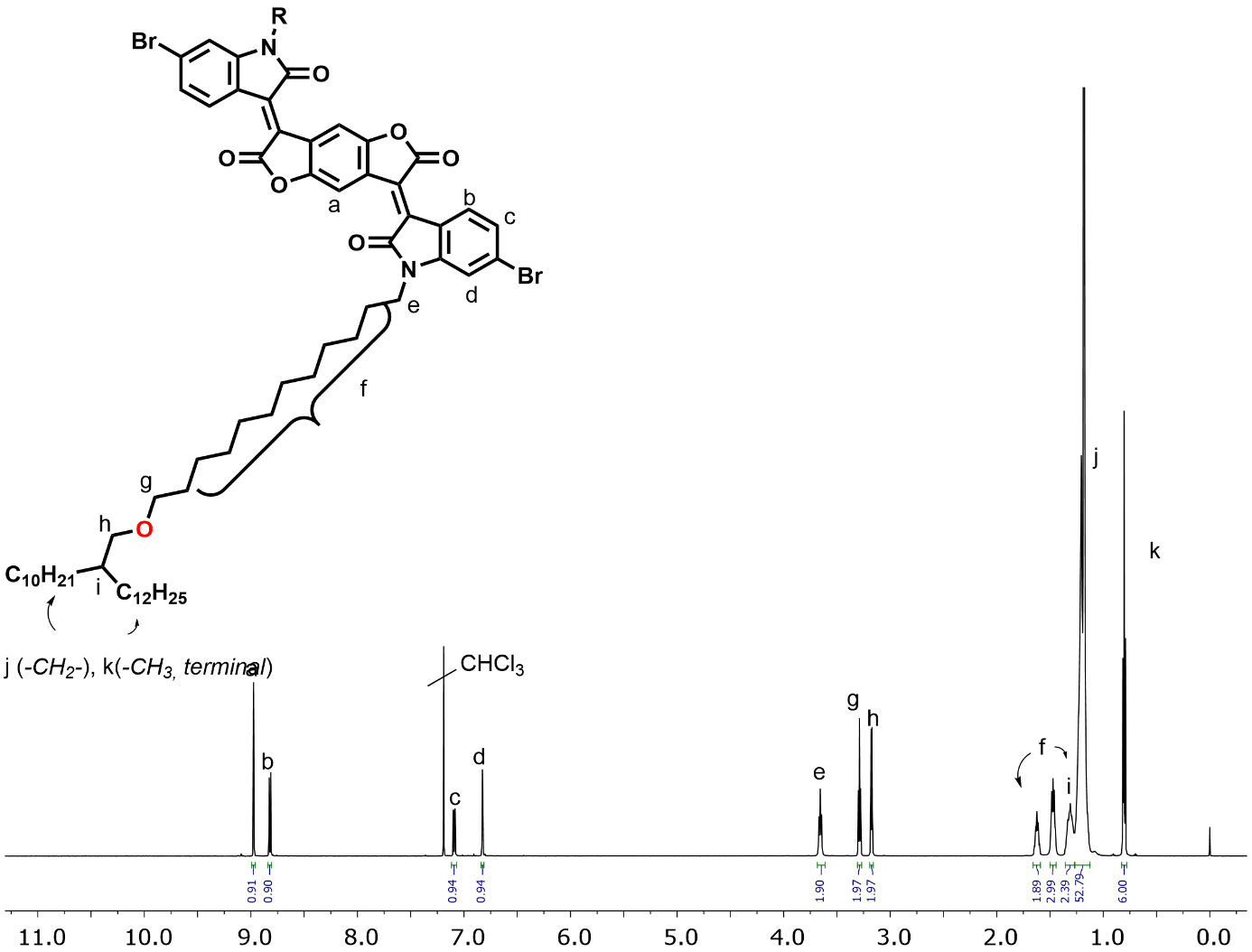


**Figure S27.** ^1^H-NMR spectrum of (**3c**) in CDCl_3_ at 298K.


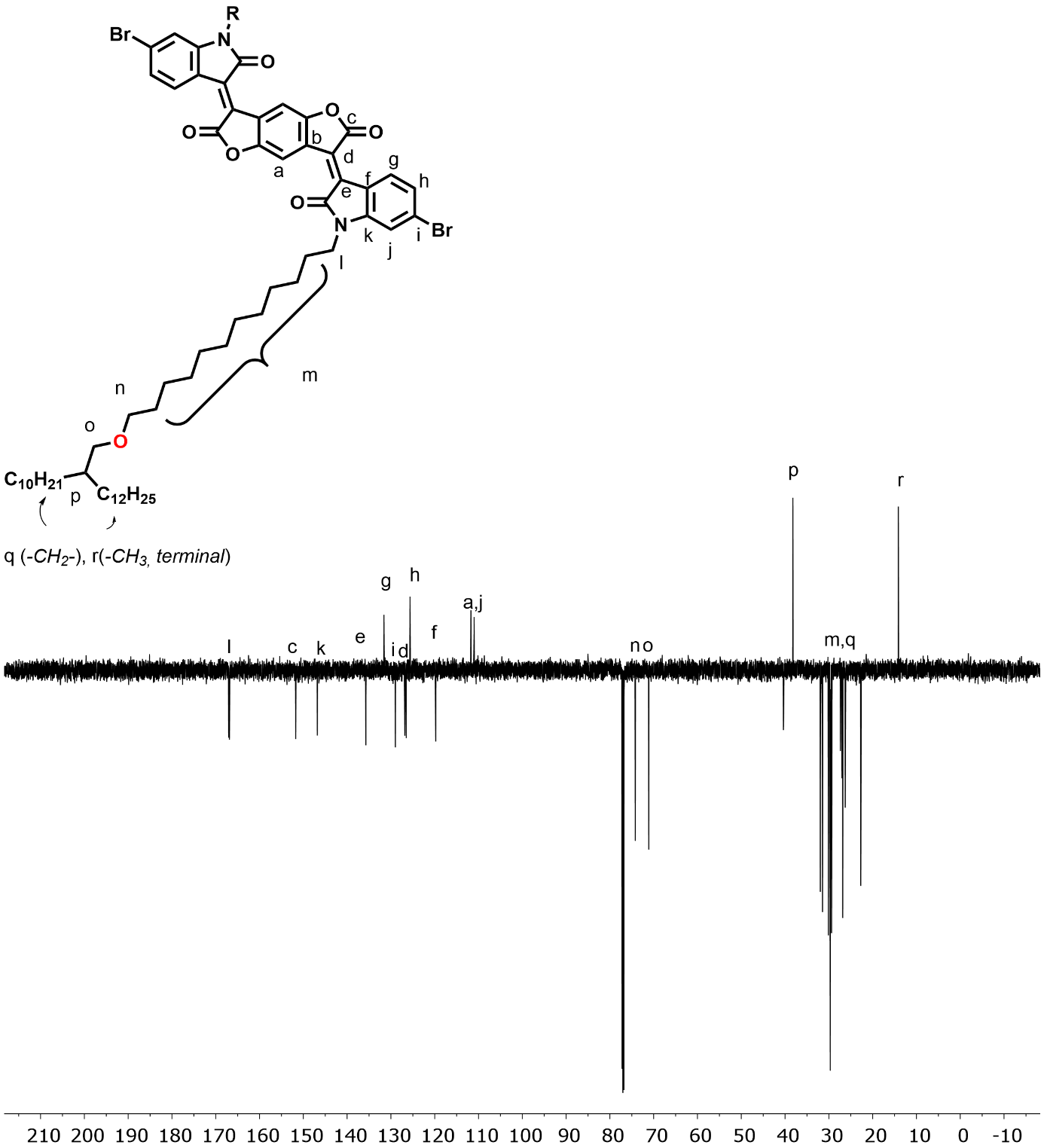


**Figure S28.** ^13^C{^1^H}-APT spectrum of (**3c**) in CDCl3 at 298K.

**4 References**

(1) Cardona, C. M.; Li, W.; Kaifer, A. E.; Stockdale, D.; Bazan, G. C. Electrochemical Considerations for Determining Absolute Frontier Orbital Energy Levels of Conjugated Polymers for Solar Cell Applications. *Advanced Materials* **2011**, *23* (20), 2367–2371.

(2) Kirby, N. M.; Mudie, S. T.; Hawley, A. M.; Cookson, D. J.; Mertens, H. D.; Cowieson, N.; Samardzic-Boban, V. A Low-Background-Intensity Focusing Small-Angle X-Ray Scattering Undulator Beamline. *Journal of Applied Crystallography* **2013**, *46* (6), 1670–1680.

(3) Ilavsky, J. *Nika*: Software for Two-Dimensional Data Reduction. *Journal of Applied Crystallography* **2012**, *45* (2), 324–328. DOI:10.1107/s0021889812004037.

(4) Beretta, D.; Bruno, P.; Lanzani, G.; Caironi, M. Reliable Measurement of the Seebeck Coefficient of Organic and Inorganic Materials between 260 K and 460 K. *Review of Scientific Instruments* **2015**, *86* (7), 075104.
